# Comprehensive investigation of predictive processing: A cross‐ and within‐cognitive domains fMRI meta‐analytic approach

**DOI:** 10.1002/hbm.26817

**Published:** 2024-08-21

**Authors:** Cristiano Costa, Rachele Pezzetta, Fabio Masina, Sara Lago, Simone Gastaldon, Camilla Frangi, Sarah Genon, Giorgio Arcara, Cristina Scarpazza

**Affiliations:** ^1^ Padova Neuroscience Center Padua Italy; ^2^ IRCCS Ospedale San Camillo Venice Italy; ^3^ Dipartimento di Psicologia dello Sviluppo e della Socializzazione Università degli Studi di Padova Padua Italy; ^4^ Dipartimento di Psicologia Generale Università degli Studi di Padova Padua Italy; ^5^ Institute for Systems Neuroscience Heinrich Heine University Düsseldorf Düsseldorf Germany; ^6^ Institute of Neuroscience and Medicine, Brain & Behaviour (INM‐7) Research Centre Jülich Jülich Germany

**Keywords:** ALE meta‐analysis, cognitive functions, domain‐general, encoding, network, predictive processing, violation

## Abstract

Predictive processing (PP) stands as a predominant theoretical framework in neuroscience. While some efforts have been made to frame PP within a cognitive domain‐general network perspective, suggesting the existence of a “prediction network,” these studies have primarily focused on specific cognitive domains or functions. The question of whether a domain‐general predictive network that encompasses all well‐established cognitive domains exists remains unanswered. The present meta‐analysis aims to address this gap by testing the hypothesis that PP relies on a large‐scale network spanning across cognitive domains, supporting PP as a unified account toward a more integrated approach to neuroscience. The Activation Likelihood Estimation meta‐analytic approach was employed, along with Meta‐Analytic Connectivity Mapping, conjunction analysis, and behavioral decoding techniques. The analyses focused on prediction incongruency and prediction congruency, two conditions likely reflective of core phenomena of PP. Additionally, the analysis focused on a prediction phenomena‐independent dimension, regardless of prediction incongruency and congruency. These analyses were first applied to each cognitive domain considered (cognitive control, attention, motor, language, social cognition). Then, all cognitive domains were collapsed into a single, cross‐domain dimension, encompassing a total of 252 experiments. Results pertaining to prediction incongruency rely on a defined network across cognitive domains, while prediction congruency results exhibited less overall activation and slightly more variability across cognitive domains. The converging patterns of activation across prediction phenomena and cognitive domains highlight the role of several brain hubs unfolding within an organized large‐scale network (Dynamic Prediction Network), mainly encompassing bilateral insula, frontal gyri, claustrum, parietal lobules, and temporal gyri. Additionally, the crucial role played at a cross‐domain, multimodal level by the anterior insula, as evidenced by the conjunction and Meta‐Analytic Connectivity Mapping analyses, places it as the major hub of the Dynamic Prediction Network. Results support the hypothesis that PP relies on a domain‐general, large‐scale network within whose regions PP units are likely to operate, depending on the context and environmental demands. The wide array of regions within the Dynamic Prediction Network seamlessly integrate context‐ and stimulus‐dependent predictive computations, thereby contributing to the adaptive updating of the brain's models of the inner and external world.

## Graphical abstract

Predictive processing units operate within a cognitive domain‐general network encompassing bilateral insula, frontal gyri, claustrum, parietal lobules, and temporal gyri. The role played at a cross‐domain, multimodal level by the anterior insula, as evidenced by the conjunction and Meta‐Analytic Connectivity Mapping analyses, places it as the major hub of the Dynamic Prediction Network.

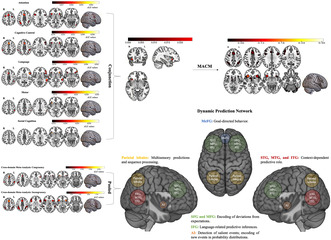

**Practitioner Points**

- Predictive processing relies on a domain‐general, large‐scale network encompassing bilateral insula, frontal gyri, claustrum, parietal lobules, and temporal gyri. Predictive processing units are likely to operate within these hubs, depending on the context and environmental demands.
- Assuming a heterarchical organization of prediction hubs would allow for the integration of context‐ and stimulus‐dependent predictive computations, contributing to the updating of the brain's models of the world.
- Investigating predictive processing across cognitive domains, this study offers insights into how this framework bridges the integrated nature of cognitive functions across well‐established domains, going beyond the conventional boundaries of mental terms.

## 1 INTRODUCTION

Cognitive processes involve complex functional brain organization and fine dynamic system‐level integration (Shine et al., 2019). However, how the activity within large‐scale networks links to cognitive functioning remains an open question (Cole et al., 2014; Shine et al., 2019). Among the theoretical models proposed to explain cognitive processes and their neural correlates over the last decades, since Rao and Ballard (1999) seminal work and up to recent theoretical models (Friston, 2003; Friston et al., 2017; Keller & Mrsic‐Flogel, 2018; Owens et al., 2018; Pezzulo et al., 2021) and empirical findings (Deco et al., 2011; Malekshahi et al., 2016; Rauss et al., 2011; Stefanics et al., 2018, 2019), predictive processing (PP) (Clark, 2013; Friston, 2010; Knill & Pouget, 2004) has emerged as a predominant theoretical framework in sensorimotor, cognitive, computational, and affective neuroscience. To date, several theories and models have been proposed within the umbrella term of “PP” (see, e.g., Aitchison & Lengyel, 2017; Euler, 2018; Nave et al., 2020; van Elk, 2021). For clarity, this work will refer to Andy Clark's general view (Clark, 2013, 2015). This theoretical perspective traces its roots back to Helmholtz's idea that perception is a process of probabilistic, knowledge‐driven, inference (Hohwy, 2013). Moving on from Kantian‐influenced views (Swanson, 2016), modern formulations of PP suggest that the information flow, modeled as a multilevel hierarchical generative model, involves higher‐level units capturing the statistical structure of observed inputs at lower‐level units, by schematically recapitulating the causal matrix responsible for that structure (Brown et al., 2011; Friston, 2010; Friston & Stephan, 2007; Lee & Mumford, 2003; Rao & Ballard, 1999). Within this framework, prediction error minimization serves as the driving force behind learning, action‐selection, recognition, and inference (Auksztulewicz & Friston, 2016; Clark, 2013; Den Ouden et al., 2012; Dołęga & Dewhurst, 2021; Feldman & Friston, 2010; Friston et al., 2017; Knill & Pouget, 2004; Walsh et al., 2020; Watanabe et al., 2018).

The natural question that arises is how predictive processes are computed at the neural level. Recent neurophysiological perspectives (Keller & Mrsic‐Flogel, 2018; Walsh et al., 2020) propose that these processes are likely to be implemented in an inferential hierarchy consisting of either two or three functionally distinct neural subpopulations (units). As per Walsh et al. (2020), expectation units convey expected sensory states downward and laterally within the processing hierarchy, and error units transmit prediction error signals upward and laterally. Keller and Mrsic‐Flogel (2018) propose a distinction among error units, delineating a comparator circuit that computes the prediction error between sensory input and predictions, and a modulating signal that establishes the precision of the prediction error. PP models describe communication between units, or neurons (Clark, 2013; Friston, 2010; Lee et al., 2021), but as this framework gained increasing evidence and support, recent literature linked these units to activity in discrete brain regions (e.g., Kilner et al., 2007; Pajani et al., 2017; Seth et al., 2012). These models also support that PP can offer insights into the emergence of the representational content of psychological phenomena, such as surprise or expectation, at the macroscale (see Lee et al., 2021 for an elegant discussion). However, most of these models depict PP units to operate in segregated hubs. Despite the longstanding assumption that cognitive functions reflect the activity and co‐activity of individual brain areas, it is well‐established that cognitive functioning arises from the dynamic interactions of distributed brain areas operating as a coherent whole in large‐scale networks that fluidly adapt to changing environmental demands (Bressler & Menon, 2010; Raichle, 2009; Shine et al., 2019).

To date, some attempts have been made to meta‐analytically investigate where PP units are likely to operate in the brain, and frame predictive mechanisms within a cognitive domain‐general network perspective. Siman‐Tov et al. (2019) conducted an Activation Likelihood Estimation (ALE) meta‐analytic investigation focused on three functional domains: action perception, language, and music, all inherently involving prediction. Results revealed significant convergence in cortical and subcortical clusters, including bilateral anterior insula, inferior frontal gyrus, and ventral premotor cortex; right pre‐supplementary motor area, middle frontal gyrus, and supramarginal gyrus; and left posterior superior temporal sulcus, caudate, and cerebellar lobule VII. As stated by the authors, this combination of brain regions is reminiscent of the neuroanatomical foundations of several neural functions, such as motor control, implicit learning, attention, and social cognition, suggesting how the presented network is implicated in diverse cognitive processes in a predictive domain‐general fashion. In a second ALE meta‐analysis, Ficco et al. (2021) categorized contrasts from each experiment into two conditions: prediction violation and prediction encoding. A third condition, general prediction, was created by merging the datasets of the other two conditions. The ALE results revealed convergence across tasks targeting PP in a set of cortical regions, such as the left inferior frontal gyrus and left insula, in both the prediction violation and general prediction condition. However, no convergence was found in the encoding condition. In addition, through Seed‐Voxel Correlations Consensus, a meta‐analytic connectivity method, the authors identified a large, bilateral predictive network resembling networks involved in task‐driven attention and task execution. In a third study, Corlett et al. (2022) employed the Multi‐level Kernel‐based Density meta‐analysis method to investigate the neural implementation of human prediction errors, providing insights into the neural mechanisms of domain‐general prediction errors in various domains such as reward, punishment, action, cognition, and perception. The study identified several brain regions associated with prediction errors, including the midbrain, dorsal and ventral striatum, thalamus, amygdala, insula, claustrum, prefrontal cortex, parietal cortex, precuneus, occipital cortex, and posterior and anterior cingulate. These studies collectively contribute valuable insights supporting the hypothesis that predictive processes rely on a domain‐general, large‐scale network. However, to date, no previous study has addressed the existence of a predictive network encompassing all well‐established cognitive domains. This could in turn provide support to the notion that the neuroarchitecture does not respect the conventional boundaries of mental terms (Pessoa et al., 2022; Poldrack & Yarkoni, 2016), and that PP bestows a compelling framework for moving toward a more integrated approach to neuroscience.

To fill this gap in the literature, the current work aims to meta‐analytically summarize neuroimaging findings capturing predictive processes across multiple cognitive domains, formally and traditionally conceptualized within the boundaries of mental terms (i.e., cognitive control, attention, motor, language, social cognition, and memory) (American Psychiatric Association, 2013; Pessoa et al., 2022). Brain regions where PP units are likely to operate will be explored by investigating prediction phenomena's macroscopic correlates (i.e., by leveraging prediction incongruency and prediction congruency conditions, see Section 2.3) and in a phenomena‐independent fashion (i.e., by merging prediction incongruency and congruency datasets, which will highlight regions consistently activated in both prediction conditions). To this end, multiple ALE meta‐analyses will be conducted (Eickhoff et al., 2009, 2016; Turkeltaub et al., 2012), regardless of task typology (which are mainly of the types “Congruent—Incongruent,” “Predictable—Unpredictable,” “Valid cue—Invalid cue,” “Standard–Deviant,” see Supplementary Information B) and stimuli used in individual studies, on data clustered as prediction incongruency, prediction congruency, and phenomena‐independent prediction. This comprehensive analysis plan will be adopted for each considered cognitive domain (cognitive control, attention, motor, language, social cognition) and by collapsing all cognitive domains into a single, cross‐domain dimension.

Building upon previous meta‐analyses, the present study will introduce a multi‐faceted approach. In addition to the ALE technique, results will be extended through the application of the Meta‐Analytic Connectivity Mapping (MACM) method, the minimum conjunction analysis, and the behavioral decoding, to ensure a comprehensive investigation of PP within‐ and cross‐cognitive domain.

## 2 MATERIALS AND METHODS

### 2.1 Pre‐registration

The protocol for the present meta‐analysis has been registered in the Open Science Framework (OSF) register (10.17605/OSF.IO/CZ5HN).

### 2.2 Study selection

An in‐depth literature search was conducted following the updated PRISMA Guidelines (Page et al., 2021) and the guidelines for neuroimaging meta‐analysis (Müller et al., 2018). The PRISMA Checklist and the Checklist for neuroimaging meta‐analysis are available in the Supplementary Information C and D, respectively. Eligible studies were identified by searching the Pubmed, Embase, and PsycINFO bibliographic databases from inception until the 30th of December 2022. The search utilized combinations of database‐specific terms such as “fMRI,” “Prediction,” “Prediction error,” “Surpris*,” and “Violat*,” along with domain‐specific terms (the complete search strings are available in Supplementary Information A S1).

Inclusion criteria encompassed studies that: (a) employed fMRI or PET; (b) focused on healthy participants of age >18 years old; (c) involved at least five participants; (d) provided 3D coordinates of peak activations within the stereotactic Montreal Neurological Institute (MNI) or Talairach space; (e) employed whole brain analysis; and (f) conducted a direct comparison of brain activation between conditions within the experimental task involving predictive processes. As for the exclusion criteria, studies were excluded if they did not employ a relevant task (i.e., frameable within the PP framework), a relevant contrast (as a control condition), or fell in‐between two cognitive domains (i.e., multi‐domain tasks). Reviews and meta‐analyses were also excluded. Studies already included in Ficco et al. (2021) and Siman‐Tov et al. (2019) were collected beforehand and added to the ones retrieved from the databases before duplicates removal. Screening and selection of studies were conducted by five authors (CC, RP, FM, SL, and SG), with data extraction randomly double checked by the same authors. Conflicts were resolved through pairwise discussions until a consensus was reached.

### 2.3 Data extraction

The following information was extracted from each paper: (a) number of participants; (b) cognitive domain; (c) modality of task stimuli presentation (e.g., visual or auditory); (d) type of task and stimuli; (e) contrast(s); (f) brain activation coordinates for the direct comparison between task conditions; (g) source of coordinates (where in the paper the coordinates were reported, e.g., Table x, Figure y, etc.); (h) statistics; (i) task nature (i.e., active or passive, indicating whether a behavioral performance was required); (l) predictability of the condition; (m) presence of a violation of the established expectancy; and (*n*) whether the predictability was explicitly stated within the task's information or rather it was implicit (Supplementary Information B). Five authors (CC, RP, FM, SL, and SG) independently extracted the data.

Datasets for analyses were created by clustering studies and experiments according to the cognitive domain categorization, with a minimum threshold of 17 experiments set to maintain adequate power for each meta‐analysis (Eickhoff et al., 2016). Studies falling under cognitive domains where the minimum threshold could not be reached were included in the cross‐domain meta‐analysis only. This approach resulted in the creation of the following datasets, which subsequently entered the analyses: cognitive control, attention, motor, language, social cognition, and cross‐domain (i.e., all the above domains plus memory, music, and pain, which did not reach the minimum threshold of 17 experiments). Additionally, each dataset was further divided into two sub‐datasets based on the typology of prediction phenomena (i.e., prediction congruency or prediction incongruency). In total, 18 datasets were created (see Supplementary Information A S4 for the number of included experiments for each dataset).

As previously mentioned, predictive computations occur at the unit level. To meta‐analytically summarize where these units are likely to operate, one should operationalize the selection of contrasts for inclusion in the meta‐analyses so that the macroscopic activity is likely reflective of underlying predictive processes. Therefore, the present work focuses on prediction congruency and prediction incongruency conditions. Following Clark's (2013, 2015) view and Lee et al.'s (2021) discussion of PP neural implementation and associated psychological states, prediction congruency would be reflective of the process through which the brain represents information from the external world or internal states that match predictions. Conversely, prediction incongruency would be reflective of a situation where the predictions generated by the brain do not align with incoming sensory information. To illustrate how this guided the selection process if a study implements an oddball task and reports activation coordinates for Standard > Deviant and Deviant > Standard contrasts, the former would be included in the prediction congruency datasets, while the Deviant > Standard contrast would be included in the prediction incongruency datasets.

It is crucial to note that when the actual sensory input deviates from the brain's predictions, it does not only generate a prediction error but also triggers a corrective adjustment in the internal model to better reflect the true state of the world. It is reasonable to assume that the neural correlates of this process are captured by activations included in the prediction incongruency datasets, thereby introducing potential confounding effects that are challenging to regress out due to the intrinsic nature of input data for an ALE meta‐analysis.

### 2.4 ALE meta‐analyses

The ALE method aims at determining the above‐chance convergence of activation probabilities between experiments (Eickhoff et al., 2016). The algorithm follows a four‐step structure. Step 1: modeling a Gaussian kernel for each activation peak, treating these as fixed‐effects within each study, whereas studies are treated as random‐effects. The width of the kernels accommodates between‐subject and between‐lab variations, addressing spatial uncertainty based on the participants' sample size in each study. Step 2: calculating a modeled activation map for each study, unifying all the modeled peaks. Step 3: obtaining ALE maps by computing the union of activation probabilities for each voxel. Step 4: testing for statistical significance by comparing the ALE scores of the obtained union map with a null‐distribution of ALE scores reflecting the random spatial association between studies, and applying a correction for multiple comparisons.

Stereotactic coordinates for the ALE meta‐analyses were extracted from the studies. The ALE algorithm was used as implemented in GingerALE 3.0.2 software (Eickhoff et al., 2009, 2012; Turkeltaub et al., 2012). Coordinates in the Talairach space were converted into the MNI 152 standard space using the GingerALE foci converter tool. Statistical significance was assessed and corrected for multiple comparisons using a cluster‐based method (Eickhoff et al., 2012, 2016): cluster‐forming threshold *p* < .001; cluster level Family Wise Error correction *p* < .05, 5000 permutations. In cases where one study had multiple contrasts reflecting the effect of interest in each condition (e.g., two contrasts reflecting different aspects of incongruency), their respective coordinates were merged to avoid duplicating participants in the analyses (Müller et al., 2018). ALE meta‐analyses were performed for all 18 datasets.

### 2.5 Conjunction and contrast analysis

Conjunction between the prediction phenomena‐independent ALE maps of each cognitive domain was carried out through SPM12's *ImCalc* function (Wellcome Trust Centre for Neuroimaging, London) by calculating a voxel‐wise minimum statistic (Nichols et al., 2005). Computationally, this is equivalent to determining the intersection between the thresholded meta‐analyses results. The results were thus significant in individual analyses at a corrected *p* < .05. The resulting area was anatomically labeled by reference to probabilistic cytoarchitectonic maps of the human brain (Eickhoff et al., 2005, 2007).

To assess the differences between cross‐domain prediction incongruency and prediction congruency, the voxel‐wise difference between the ensuing ALE maps was computed (Eickhoff et al., 2011). Contrast analysis involves comparing two ALE datasets by generating two ALE contrast images through direct subtraction of one input image from the other. As this ALE subtraction image does not account for differences between the studies, to address this problem and correct for study sizes, GingerALE employs a strategy of creating simulated data. This entails pooling the foci datasets and randomly dividing them into two new groupings of the same size as the original datasets. An ALE image is then generated for each new dataset, subtracted from the other, and compared to the true data. Following *n* permutations, a voxel‐wise *p*‐value image is obtained, showing where the values of the true data sit within the distribution of values in that voxel (Eickhoff et al., 2011, 2012). Differences of convergence were assessed by means of an uncorrected *p* < .01, 10,000 permutations, and a cluster threshold of 200 mm^3^.

### 2.6 Meta‐analytic connectivity mapping

Sleuth v3.0.4 and GingerALE v3.0.2 software were used to perform the Meta Analytic Connectivity Mapping. MACM delineates the patterns of co‐activation across numerous studies by leveraging neuroimaging databases, and generating data‐driven functional connectivity maps based on a predefined seed region (Langner et al., 2014). For the present analysis, the seed region was defined as the area of conjunction among all cognitive domain‐based meta‐analyses. The BrainMap database (http://www.brainmap.org/) was used, containing, at the time of assessment, coordinates of reported activation foci and associated meta‐data from nearly 17,000 neuroimaging experiments. The inclusion criteria for the present analysis involved whole‐brain neuroimaging studies reporting at least one activation focus within the seed region in a healthy population. Exclusion criteria encompassed studies investigating differences in age, gender, handedness, training effects, or clinical populations. Subsequently, an ALE meta‐analysis was performed on the extracted experiments to test for spatial convergence across all reported foci. The statistical thresholds applied were consistent with the other ALE analyses (i.e., cluster‐forming threshold *p* < .001; cluster level Family Wise Error correction *p* < .05, 5000 permutations). The seed region would exhibit high convergence, and convergence outside the seed region indicates other brain regions demonstrating consistent co‐activations with it, reflecting task‐based functional connectivity (Goodwill et al., 2023).

### 2.7 Behavioral decoding

Behavioral decoding was performed to explore behavioral concepts commonly associated with the activations of a seed region (Genon et al., 2018), defined, like for the MACM analysis, as the area of conjunction among all cognitive domain‐based meta‐analyses. Particularly, behavioral decoding was performed using the “behavioral domain” (BD) and “paradigm class” (PC) metadata as assigned in the BrainMap database (Laird et al., 2009). A description of the behavioral processes covered by the BrainMap PC taxonomy can be found athttps://brainmap.org/taxonomy/paradigms/. BDs include the main categories “cognition, action, perception, emotion, interoception,” along with their respective subcategories. PCs categorize the specific tasks employed. To characterize the functional profile of the seed region, quantitative “forward inference” and “reverse inference” approaches were employed (Genon et al., 2017).

In the forward inference approach, a region's functional profile is assessed by identifying taxonomic labels for which the probability of finding activation in the respective region is significantly higher than finding activation for that label across the whole database by chance. Significance was determined using a binomial test (*p* < .05, corrected for multiple comparisons using Bonferroni's method; Clos et al., 2013; Genon et al., 2017; Rottschy et al., 2013). This involves testing whether the conditional probability of activation in a particular region given a particular label [P(Activation|Task)] is higher than the baseline probability of activating this particular region [P(Activation)].

In the reverse inference approach, a region's functional profile is determined by identifying the most likely BDs and PCs given activation in a particular cluster, that is, the likelihood P(Task|Activation). This likelihood is derived from P(Activation|Task) as well as P(Task) and P(Activation) using Bayes' rule. Significance (at *p* < .05, corrected for multiple comparisons) was assessed by means of a *χ* ^2^ test. In summary, forward inference assesses the probability of activation given a behavioral label, whereas reverse inference assesses the probability of each behavioral label given an activation.

Additionally, the association tool implemented in the Neurosynth database (https://neurosynth.org/locations/) was used, entering the peak coordinate of the seed region. The ensuing associations' table provides information about the relationship between activation at a given voxel and, among others information available in Neurosynth, the *z*‐score value obtained at the current voxel in the “association test” meta‐analysis map for the corresponding term. The analysis concentrated on terms whose associations were deemed very strong, with a *z*‐score ≥3.

### 2.8 Additional analyses

Noticing a remarkable resemblance between the cross‐domain prediction phenomena‐independent meta‐analytic results and MACM ones, it was decided to formally test the intersection between the two ensuing ALE maps. To do so, the conjunction approach was employed, following the same method outlined in Section 2.5.

As per pre‐registration, two additional analyses were conducted. First, the meta‐analysis by Siman‐Tov (Siman‐Tov et al., 2019) was replicated, using the ALE approach implementing the same statistical threshold (false discovery rate, *q* = 0.05) and minimum cluster size (200 mm^3^) (Supplementary Information A S5). Second, domain‐specific ALE results were replicated using the Seed‐Based D Mapping (SDM) approach. Details of the methods, results, between‐study heterogeneity (*I* ^2^), and publication bias (assessed through the Excess Significance Test) can be found in Supplementary Information A S6. The inclusion of the SDM approach was deliberately chosen to establish a comprehensive validation framework for the ALE results, given the latter method's shortcomings (e.g., ALE relies on reported coordinates of activation peaks rather than statistical maps, potentially leading to information loss that may not capture the full extent of activation patterns (Salimi‐Khorshidi et al., 2009)). By incorporating the SDM approach, the goal was to enhance the robustness of the findings through a methodologically diverse perspective. This dual‐method approach allows for rigorous cross‐validation of activation patterns, mitigating the risk of method‐specific biases and reinforcing the reliability of the identified neural substrates associated with the studied phenomenon.

## 3 RESULTS

### 3.1 Included studies

**FIGURE 1:**
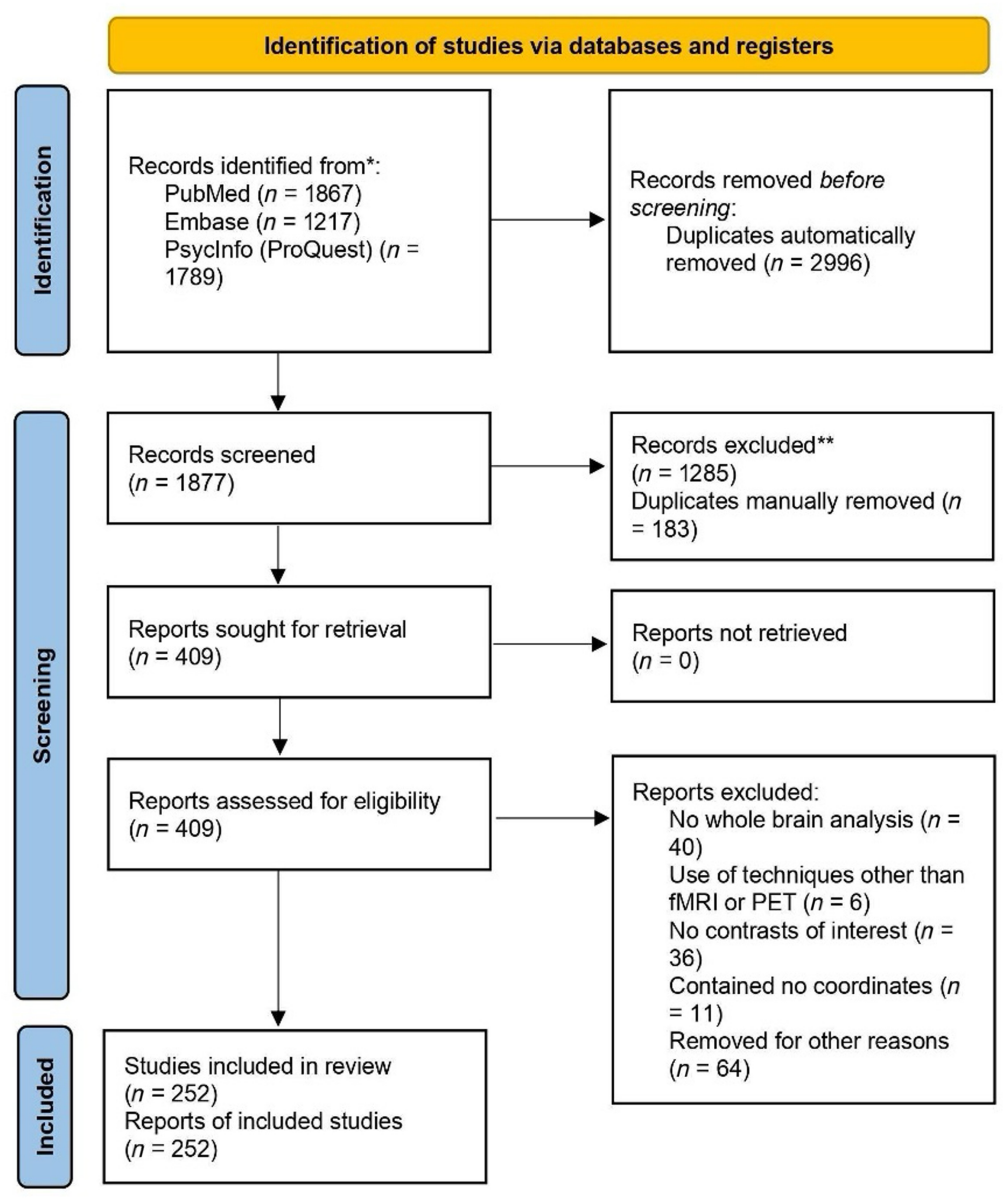
PRISMA flowchart illustrating the selection process of the present meta‐analysis.

The search produced 4873 entries (i.e., *n* = 1867 from PubMed, *n* = 1217 from Embase, *n* = 1789 from PsychInfo). After excluding 2996 entries based on title and abstract for not meeting the general inclusion criteria, the remaining 1877 entries were retrieved. Following the PRISMA guidelines, a total of 252 experiments were ultimately included in the meta‐analysis (see Figure 1 for the PRISMA Flow Chart). A detailed description of the experiments for each included study, encompassing the number of participants, cognitive domain, fMRI tasks and stimuli, significant contrast(s), foci and reference space, statistics, and the predictive characteristics of the tasks, can be found in Supplementary Information B.

### 3.2 ALE estimation

#### 3.2.1 Cross‐domain ALE

**FIGURE 2:**
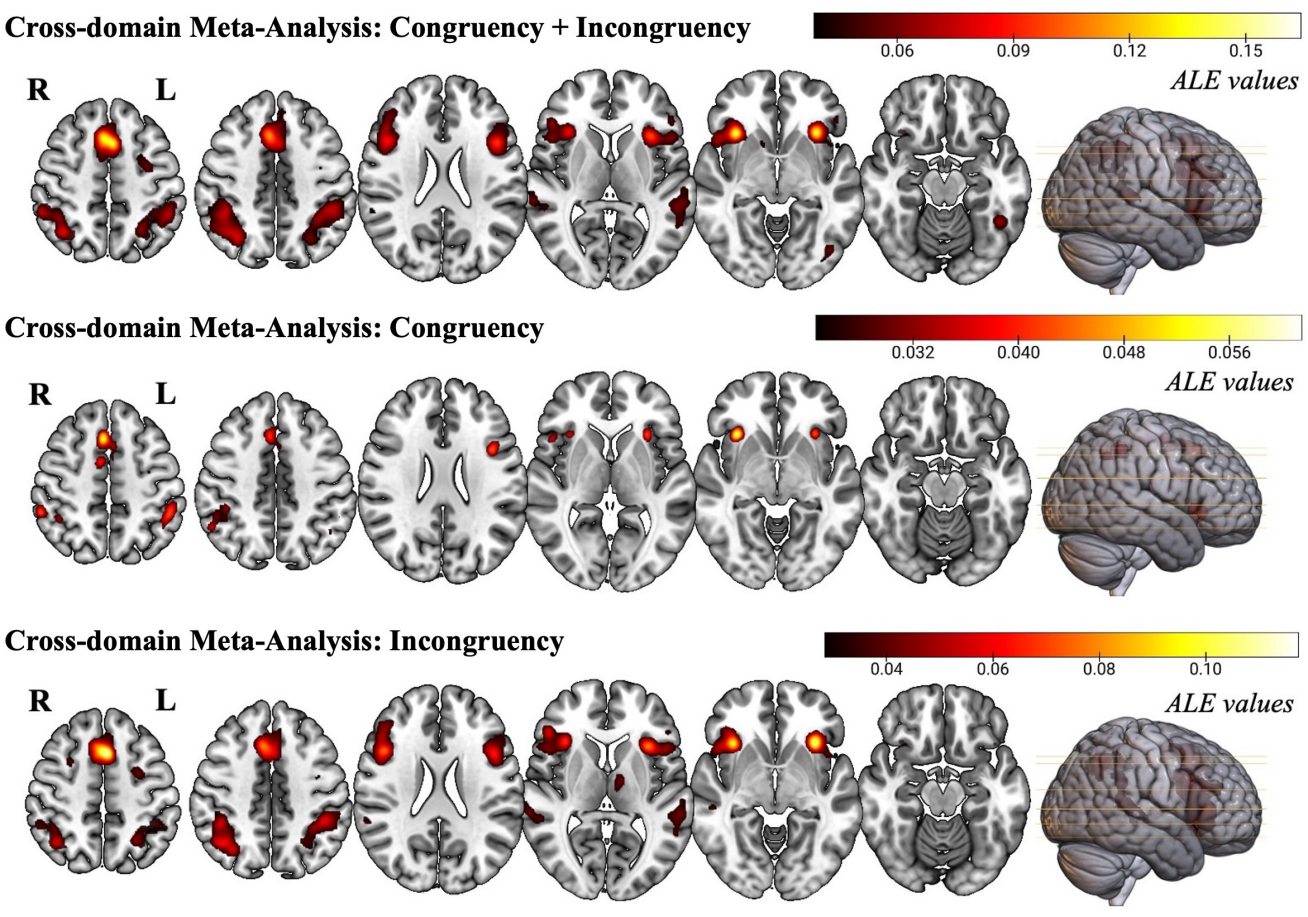
Clusters of consistent activations for the cross‐domain prediction phenomena‐independent meta‐analysis (top), cross‐domain prediction incongruency meta‐analysis (middle), cross‐domain prediction congruency meta‐analysis (bottom). Images are in radiological convention (R = Right; L = Left). ALE, Activation Likelihood Estimation.

All 252 experiments entered the cross‐domain prediction phenomena‐independent meta‐analysis, contributing 5950 participants. Significant consistent activation was found in seven clusters (Table 1, Figure 2, top row). These clusters encompassed the insula, inferior frontal gyrus, middle frontal gyrus, precentral gyrus, claustrum, caudate, lentiform nucleus, extra‐nuclear, and superior frontal gyrus (Cluster 1); the inferior frontal gyrus, precentral gyrus, insula, middle frontal gyrus, claustrum, and extra‐nuclear (Cluster 2); the inferior parietal lobule, precuneus, superior temporal gyrus, superior parietal lobule, angular gyrus, supramarginal gyrus, and middle temporal gyrus (Cluster 3); the medial frontal gyrus, superior frontal gyrus, and cingulate gyrus (Cluster 4); the inferior parietal lobule, superior parietal lobule, supramarginal gyrus, precuneus, middle temporal gyrus, and superior temporal gyrus (Cluster 5); the superior temporal gyrus, middle temporal gyrus, transverse temporal gyrus, and inferior temporal gyrus (Cluster 6) and the fusiform gyrus, inferior occipital gyrus, declive, inferior temporal gyrus, and middle occipital gyrus (Cluster 7).

**TABLE 1:** Cross‐domain prediction phenomena‐independent ALE results; cross‐domain prediction congruency Activation Likelihood Estimation (ALE) results; cross‐domain prediction incongruency ALE results.

Cluster	MNI coordinates	*Z*‐values	Region
Cross‐domain: Prediction phenomena‐independent			
1	34 22 −2	11.998	Claustrum
1	46 16 24	7.324	Inferior frontal gyrus
1	46 18 0	7.092	Insula
1	48 30 6	5.339	Inferior frontal gyrus
1	48 10 36	5.129	Precentral gyrus
1	40 38 26	5.083	Middle frontal gyrus
1	12 12 −2	3.775	Caudate
1	20 16 −10	3.672	Lentiform nucleus
2	−32 22 −2	11.409	Claustrum
2	−44 12 26	8.045	Inferior frontal gyrus
2	−30 −6 56	5.006	Precentral gyrus
2	−50 32 8	4.552	Inferior frontal gyrus
2	−48 32 −4	4.098	Inferior frontal gyrus
2	−44 12 −8	3.875	Insula
2	−52 10 −8	3.313	Superior temporal gyrus
2	−40 40 4	3.247	Inferior frontal gyrus
3	32 −64 44	6.520	Precuneus
3	42 −44 42	6.324	Inferior parietal lobule
3	54 −44 50	6.013	Inferior parietal lobule
3	64 −34 10	5.315	Superior temporal gyrus
3	54 −42 10	4.758	Superior temporal gyrus
3	52 −40 10	4.729	Superior temporal gyrus
3	52 −58 32	3.835	Superior temporal gyrus
3	56 −44 36	3.808	Supramarginal gyrus
3	58 −40 26	3.794	Inferior parietal lobule
4	−2 14 50	10.618	Superior frontal gyrus
4	4 18 48	10.580	Superior frontal gyrus
4	−4 28 40	5.296	Cingulate gyrus
4	−4 −4 58	4.129	Medial frontal gyrus
4	−4 40 46	3.836	Superior frontal gyrus
5	−48 −42 46	6.289	Inferior parietal lobule
5	−36 −48 42	5.805	Inferior parietal lobule
5	−26 −56 50	5.212	Superior parietal lobule
5	−56 −46 38	4.828	Supramarginal gyrus
5	−28 −64 44	4.828	Precuneus
5	−46 −62 26	4.037	Middle temporal gyrus
5	−40 −66 48	3.397	Inferior parietal lobule
6	−56 −38 6	5.147	Middle temporal gyrus
6	−64 −38 12	4.834	Superior temporal gyrus
6	−58 −28 12	4.783	Superior temporal gyrus
6	−60 −24 10	4.769	Superior temporal gyrus
6	−58 −50 6	4.283	Middle temporal gyrus
6	−56 −56 4	4.240	Middle temporal gyrus
6	−48 −60 0	3.429	Inferior temporal gyrus
7	−46 −50 −16	5.511	Fusiform gyrus
7	−42 −76 −8	5.199	Fusiform gyrus
7	−36 −84 −2	3.519	Inferior occipital gyrus
Cross domain: Prediction congruency			
1	4 20 48	6.408	Superior frontal gyrus
1	6 0 52	4.837	Medial frontal gyrus
1	−4 −6 58	4.691	Medial frontal gyrus
1	−4 12 48	4.279	Medial frontal gyrus
2	34 22 −2	6.776	Claustrum
2	48 18 2	4.716	Precentral gyrus
3	56 −42 50	5.238	Inferior parietal lobule
3	52 −54 44	4.366	Inferior parietal lobule
3	40 −48 48	4.026	Inferior parietal lobule
3	40 −38 44	3.963	Inferior parietal lobule
3	48 −46 44	3.713	Inferior parietal lobule
4	−50 −42 52	5.189	Inferior parietal lobule
4	−56 −46 40	4.461	Supramarginal gyrus
5	−30 24 −2	5.927	Claustrum
6	−40 10 26	5.451	Inferior frontal gyrus
Cross domain: Prediction incongruency			
1	34 22 −2	10.175	Claustrum
1	46 16 24	7.619	Inferior frontal gyrus
1	48 30 8	6.020	Inferior frontal gyrus
2	−32 22 −2	10.421	Claustrum
2	−44 6 30	7.054	Inferior frontal gyrus
2	−50 16 16	6.606	Inferior frontal gyrus
2	−44 14 22	6.518	Inferior frontal gyrus
2	−44 22 36	4.494	Precentral gyrus
2	−50 32 10	4.031	Inferior frontal gyrus
2	−38 −6 44	3.533	Precentral gyrus
2	−50 8 −8	3.462	Superior temporal gyrus
3	−50 8 −8	10.404	Superior frontal gyrus
3	−6 30 40	4.826	Cingulate gyrus
4	38 −60 46	6.991	Inferior parietal lobule
4	42 −44 44	5.904	Inferior parietal lobule
4	32 −64 44	5.833	Precuneus
4	52 −46 52	4.277	Inferior parietal lobule
5	−44 −42 44	6.170	Inferior parietal lobule
5	−36 −46 42	5.431	Inferior parietal lobule
5	−28 −56 48	4.929	Superior parietal lobule
5	−28 −62 38	3.952	Precuneus
5	−56 −42 36	3.708	Supramarginal gyrus
5	−42 −46 58	3.445	Inferior parietal lobule
6	56 −42 14	5.090	Superior temporal gyrus
6	64 −34 8	4.764	Superior temporal gyrus
6	62 −38 12	4.715	Superior temporal gyrus
6	58 −40 28	4.474	Inferior parietal lobule
6	52 −32 −2	4.094	Superior temporal gyrus
6	56 −46 34	3.946	Supramarginal gyrus
6	60 −24 2	3.704	Superior temporal gyrus
6	68 −26 6	3.649	Superior temporal gyrus
7	−64 −38 12	4.762	Superior temporal gyrus
7	−56 −52 6	4.269	Middle temporal gyrus
7	−50 −34 12	4.165	Superior temporal gyrus
7	−60 −46 16	3.256	Superior temporal gyrus
8	−28 −4 56	5.112	Middle frontal gyrus
9	−10 −10 6	4.627	Thalamus

Abbreviations: BA, Brodmann area; MNI, Montreal Neurological Institute.

The cross‐domain prediction congruency meta‐analysis (Figure 2, middle row), conducted on 134 experiments and 3258 participants, produced seven clusters of significant consistent activation (Table 1). Postcentral gyrus, which comprises 44.3% of cluster 7, emerged as the only area of activation that is not shared with the domain‐ and prediction phenomena‐independent ALE analysis.

Concerning the cross‐domain prediction incongruency dataset (Figure 2, bottom row), 175 experiments entered the analysis, contributing 4102 participants. Ten clusters showed significant consistent activation (Table 1). The largest cluster (volume = 23,360 mm^3^) encompassed the insula, inferior frontal gyrus, middle frontal gyrus, precentral gyrus, and claustrum. Of note, the smallest cluster (volume = 1272 mm^3^) belonged to the left thalamus (100%).

#### 3.2.2 Domain‐specific ALE

**FIGURE 3:**
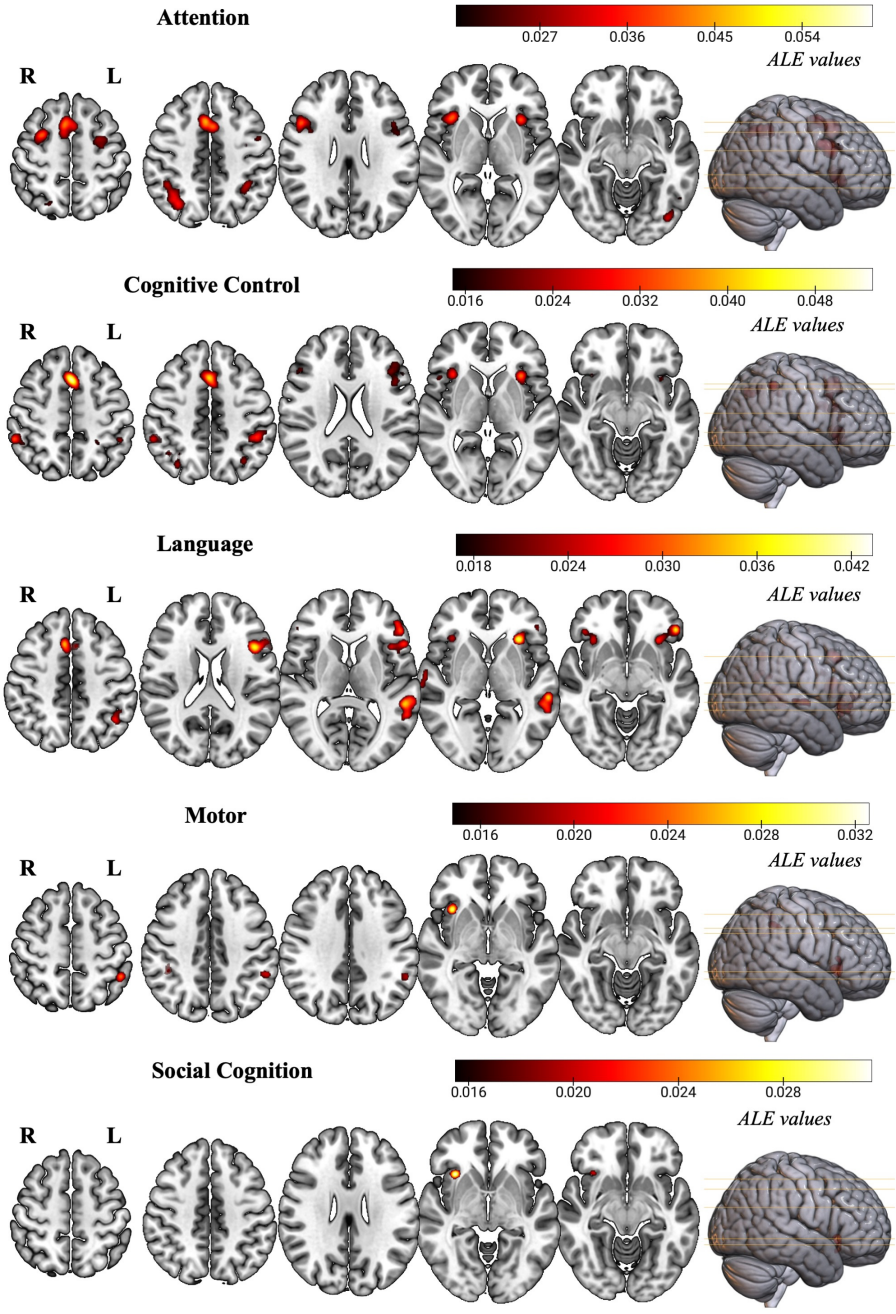
Clusters of consistent activations for each domain‐specific prediction phenomena‐independent meta‐analysis. Images are in radiological convention (R = Right; L = Left). ALE, Activation Likelihood Estimation.

Domain‐specific prediction phenomena‐independent ALE results are reported in Table 2 and graphically represented in Figure 3, while domain‐specific ALE results divided for incongruency and congruency are reported in Supplementary Information A S2 and Figure S1.

**TABLE 2:** Domain‐specific prediction phenomena‐independent Activation Likelihood Estimation results.

Cluster	MNI coordinates	*Z*‐values	Region
Prediction phenomena‐independent: Attention			
1	4 16 50	7.811	Superior frontal gyrus
1	−4 12 50	7.637	Medial frontal gyrus
2	48 6 34	5.840	Precentral gyrus
2	48 16 28	5.770	Inferior frontal gyrus
2	38 6 30	4.320	Precentral gyrus
2	56 22 32	3.459	Middle frontal gyrus
3	36 −60 46	5.354	Precuneus
3	32 −64 46	5.308	Superior parietal lobule
3	42 −46 48	4.893	Inferior parietal lobule
3	22 −66 54	3.764	Precuneus
3	46 −34 42	3.372	Inferior parietal lobule
4	36 22 0	6.812	Insula
5	−32 18 4	5.844	Claustrum
5	−34 22 −4	5.711	Insula
6	30 −6 48	3.511	Middle frontal gyrus
7	−32 −8 50	4.686	Middle frontal gyrus
7	−28 −2 54	4.324	Middle frontal gyrus
8	−40 −78 −10	4.817	Fusiform gyrus
8	−46 −50 −16	4.511	Fusiform gyrus
8	−42 −62 −14	3.625	Fusiform gyrus
8	−44 −68 −14	3.405	Fusiform gyrus
9	−36 −46 44	4.680	Inferior parietal lobule
10	−48 0 46	4.030	Precentral gyrus
10	−44 14 28	3.988	Inferior frontal gyrus
10	−46 6 30	3.730	Inferior frontal gyrus
10	−44 0 38	3.328	Precentral gyrus
11	−60 −44 16	3.879	Superior temporal gyrus
11	−56 −26 12	3.798	Superior temporal gyrus
11	−62 −38 14	3.484	Superior temporal gyrus
Prediction phenomena‐independent: Cognitive control			
1	−42 10 28	5.187	Inferior frontal gyrus
1	−40 4 30	5.030	Precentral gyrus
1	−48 18 10	4.461	Inferior frontal gyrus
1	−42 24 22	4.120	Middle frontal gyrus
1	−44 30 22	3.909	Middle frontal gyrus
1	−54 22 22	3.442	Inferior frontal gyrus
2	0 16 50	7.779	Superior frontal gyrus
3	−32 22 −2	6.770	Claustrum
4	34 20 −2	5.658	Claustrum
4	48 18 0	4.576	Insula
5	−46 −40 46	5.386	Inferior parietal lobule
5	−58 −46 40	4.859	Supramarginal gyrus
6	44 12 38	4.260	Precentral gyrus
6	50 20 28	4.242	Middle frontal gyrus
6	48 26 20	4.007	Middle frontal gyrus
6	44 14 26	3.814	Inferior frontal gyrus
7	32 −68 44	4.792	Precuneus
7	40 −56 48	3.937	Inferior parietal lobule
8	56 −42 50	6.147	Inferior parietal lobule
9	−32 −66 42	4.352	Precuneus
9	−34 −64 46	4.269	Precuneus
9	−24 −44 52	3.793	Precuneus
9	−32 −52 48	3.521	Inferior parietal lobule
Prediction phenomena‐independent: Language			
1	−30 24 −2	6.319	Claustrum
1	−48 34 −6	5.937	Inferior frontal gyrus
1	−44 16 20	5.816	Inferior frontal gyrus
1	−56 22 16	5.625	Inferior frontal gyrus
1	−50 34 8	4.956	Inferior frontal gyrus
1	−48 38 8	4.810	Inferior frontal gyrus
1	−42 16 10	4.255	Insula
1	−42 6 30	3.851	Precentral gyrus
1	−44 8 38	3.459	Middle frontal gyrus
2	−54 −40 6	6.323	Middle temporal gyrus
2	−60 −34 4	5.671	Middle temporal gyrus
2	−64 −40 14	4.941	Superior temporal gyrus
3	34 24 −4	4.434	Insula
3	42 32 −8	4.191	Inferior frontal gyrus
3	52 34 −2	3.923	Inferior frontal gyrus
3	52 36 6	3.650	Inferior frontal gyrus
4	6 20 46	5.396	Medial frontal gyrus
4	−4 16 50	4.561	Superior frontal gyrus
5	−56 −46 26	4.185	Supramarginal gyrus
5	−44 −58 26	4.164	Middle temporal gyrus
5	−46 −54 30	4.111	Superior temporal gyrus
6	−44 −56 48	4.505	Inferior parietal lobule
7	62 −12 2	4.060	Superior temporal gyrus
7	66 −20 2	4.053	Superior temporal gyrus
Prediction phenomena‐independent: Motor			
1	−50 −46 54	4.529	Inferior parietal lobule
1	−56 −44 38	3.982	Supramarginal gyrus
2	36 20 −2	5.573	Claustrum
2	34 22 10	4.103	Insula
3	42 −42 42	4.385	Inferior parietal lobule
Prediction phenomena‐independent: Social cognition			
1	32 20 −4	5.226	Claustrum
1	32 20 −16	3.800	Extra‐nuclear
1	42 20 −12	3.427	Inferior frontal gyrus

Abbreviations: BA, Brodmann area; MNI, Montreal Neurological Institute.

##### Attention

Consistent activation across attention tasks regardless of the type of prediction phenomena (*n* = 52 experiments) was found in the right superior, right and left middle, and right and left inferior frontal gyri, right and left insula, right and left precentral gyrus, right superior and right inferior parietal lobules, left superior temporal gyrus, left fusiform gyrus, and left claustrum. In the context of prediction incongruency (*n* = 44 experiments), the same areas of activation of the attention prediction phenomena‐independent analysis were found, while regarding prediction congruency (*n* = 17 experiments), consistent activation was restricted to the right superior frontal gyrus, left cingulate gyrus, left insula, left superior temporal gyrus, right precentral gyrus, and left inferior parietal lobule.

##### Cognitive control

Consistent activation across cognitive control tasks regardless of the type of prediction phenomena (*n* = 31 experiments) was found in the left superior, right, and left middle, and right and left inferior frontal gyri, right insula, right and left precentral gyrus, right and left inferior parietal lobule, right and left claustrum, and right and left precuneus. For what concerns prediction incongruency (*n* = 21 experiments), the same areas of activation of the cognitive control prediction phenomena‐independent analysis were found, while regarding prediction congruency (*n* = 15 experiments), consistent activation was restricted to right superior and right middle frontal gyri, right inferior parietal lobule, and left pyramis.

##### Language

Consistent activation across language tasks regardless of the type of prediction phenomena (*n* = 53 experiments) was found in the left superior, right and left middle and right and left inferior frontal gyri, right and left insula, left claustrum, right and left superior and left middle temporal gyri, left inferior parietal lobule, left precentral gyrus, and left supramarginal gyrus. In the case of prediction incongruency (*n* = 39 experiments), the same areas of activation of the language prediction phenomena‐independent analysis were found, while regarding prediction congruency (*n* = 32 experiments), consistent activation also extended to the left fusiform gyrus.

##### Motor

Consistent activation across motor tasks regardless of the type of prediction phenomena (*n* = 41 experiments) was found in the right insula, right and left inferior parietal lobule, right claustrum, and left supramarginal gyrus. For what concerns prediction incongruency (*n* = 18 experiments), convergence of activation was found in the right and left insula, right cingulate gyrus, right superior and left middle frontal gyri, right and left claustrum, and right cingulate, while regarding prediction incongruency (*n* = 28 experiments), consistent activation was restricted to a single cluster belonging to the left supramarginal gyrus.

##### Social cognition

Consistent activation across social cognition tasks regardless of the type of prediction phenomena (*n* = 35 experiments) was found in the right insula, right claustrum, right extra nuclear, and right inferior frontal gyrus. In the context of prediction incongruency (*n* = 29 experiments), the same areas of activation of the social cognition prediction phenomena‐independent analysis were found, while regarding prediction congruency (*n* = 18 experiments), consistent activation encompassed the right caudate.

### 3.3 Conjunction and contrast analysis

**FIGURE 4:**
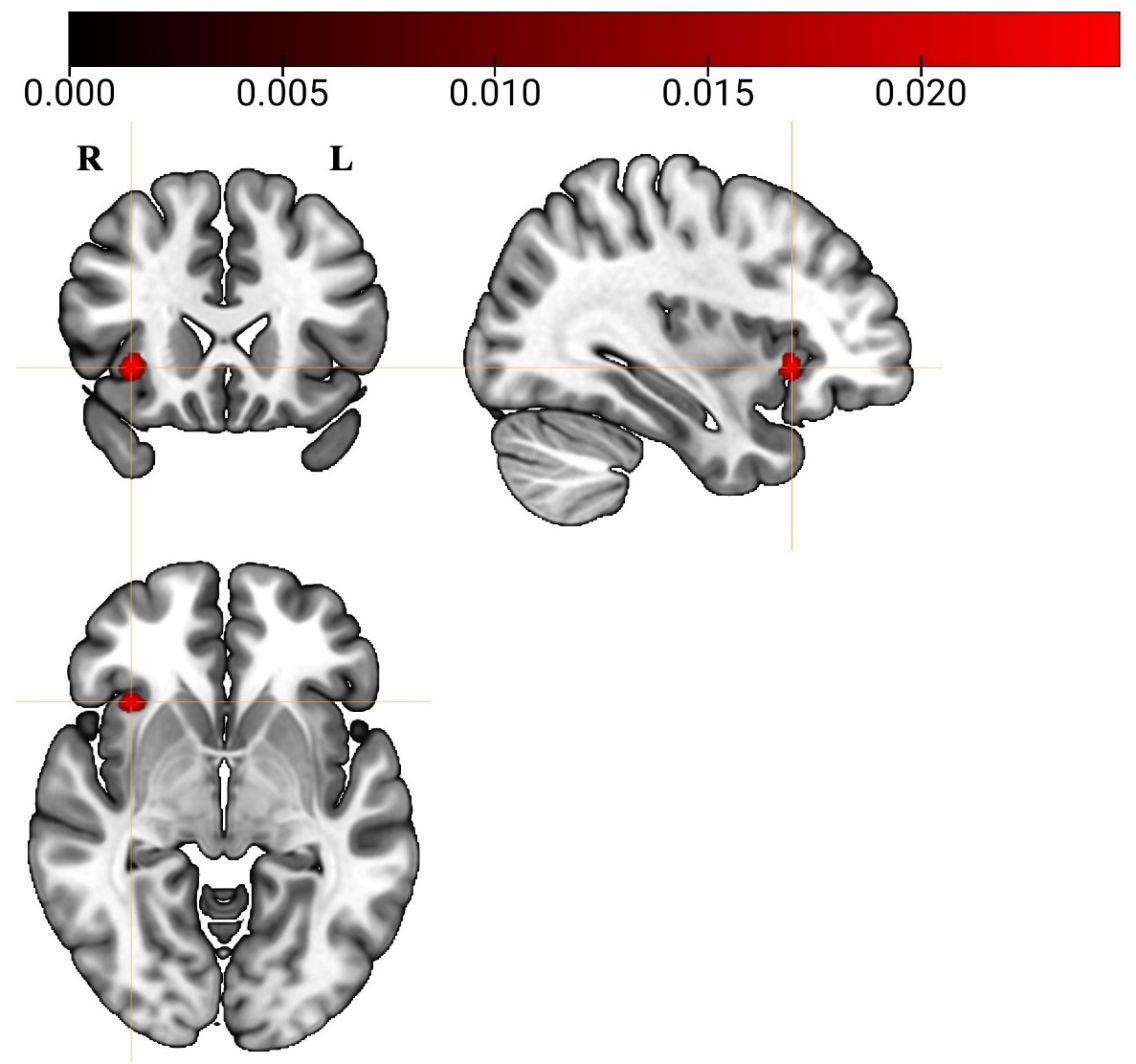
Right anterior insula emerged from the conjunction analysis across all cognitive domains investigated. Images are in radiological convention (R = Right; L = Left).

By means of a minimum conjunction analysis, a formal examination was conducted to identify brain regions consistently involved across all different types of studies within each cognitive domain under investigation (i.e., cognitive control, attention, motor, language, and social cognition), irrespective of the type of prediction phenomena. Results of this analysis demonstrate an intersection between the thresholded meta‐analyses results at the level of the right anterior insula (Figure 4).

For what concerns the differences in convergence analysis, cross‐domain prediction incongruency and congruency contributed to 175 and 134 experiments, respectively. The contrast “cross‐domain prediction incongruency > cross‐domain prediction congruency” yielded nine significant clusters encompassing the right and left insula, left superior, right and left middle and right and left inferior frontal gyri, right precentral gyrus, right inferior parietal lobule, right angular gyrus, and right middle and right superior temporal gyri (Supplementary Information A S3). The contrast “cross‐domain prediction congruency > cross‐domain prediction incongruency” produced three clusters of activations encompassing the left middle frontal gyrus, right paracentral lobule, left precentral gyrus, and left inferior parietal lobule (Supplementary Information A S3).

### 3.4 Meta‐analytic connectivity mapping

**FIGURE 5:**
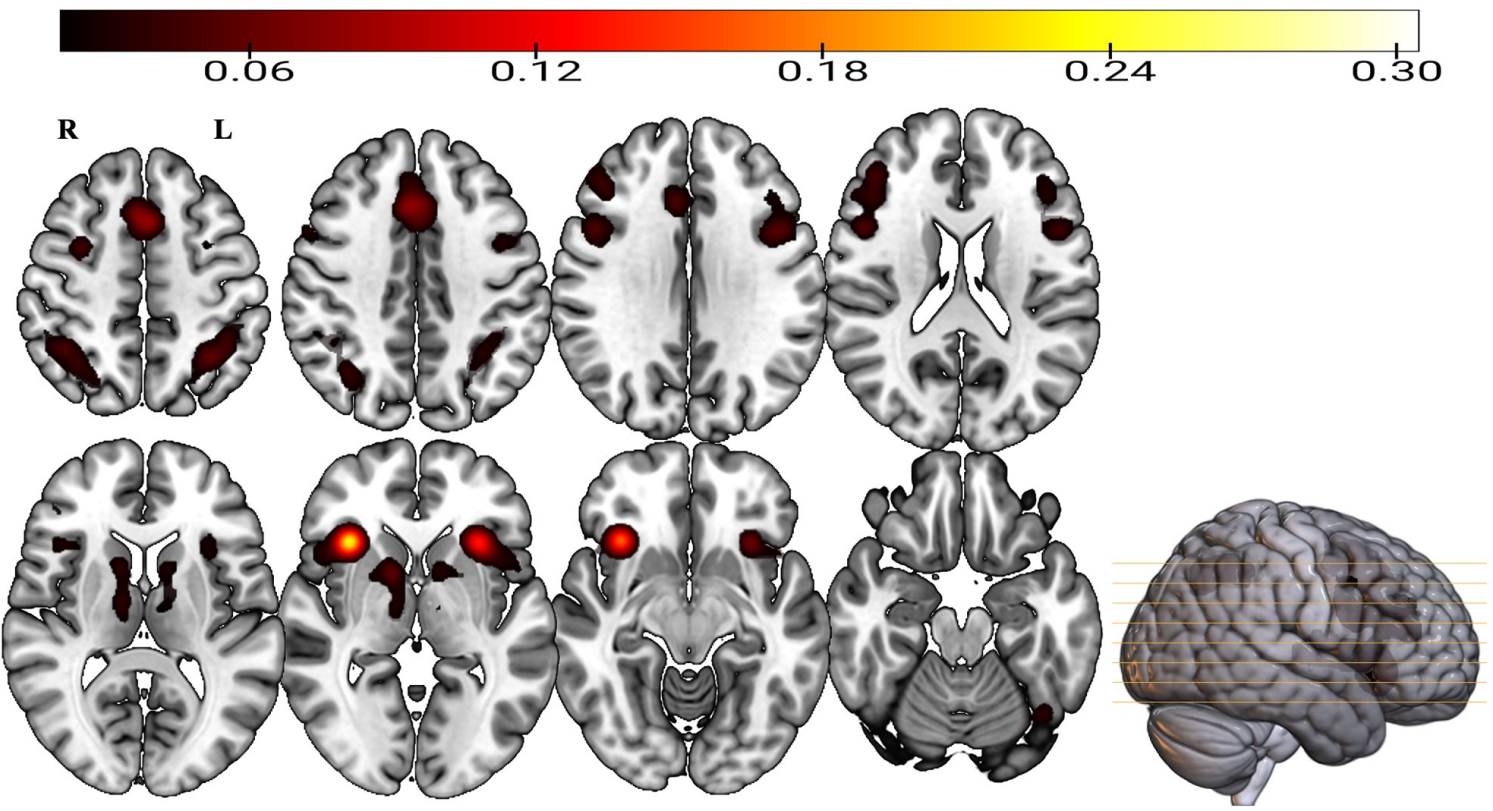
Meta‐analytic results from the Meta‐Analytic Connectivity Mapping analysis carried out on the right anterior insula. Images are in radiological convention (R = Right; L = Left).

The right anterior insula, extracted as an ROI from the minimum conjunction analysis, was used as the seed region for the MACM. BrainMap search identified 71 experiments (1189 participants) that reported at least one focus of activation within the seed. The right insula seed (cluster 1, centered at 39.1, 20.4, 9.6, volume = 24,264 mm^3^, maximum ALE value = .3044) showed significant co‐activations with seven clusters around the cingulate gyrus, thalamus, precentral gyrus, left insula, bilateral inferior parietal lobule and precuneus, and the declive (Table 3 and Figure 5).

**TABLE 3:** Meta‐analytic connectivity mapping analysis results were carried out on the right anterior insula.

Cluster	MNI coordinates			Peak ALE *p*‐value	Region
Meta‐analytic connectivity modelling					
1	34	22	−4	.304	Claustrum
1	48	10	26	.074	Inferior frontal gyrus
1	44	36	18	.048	Middle frontal gyrus
1	42	26	24	.047	Middle frontal gyrus
1	32	0	50	.047	Middle frontal gyrus
1	42	30	26	.046	Middle frontal gyrus
1	56	8	40	.032	Middle frontal gyrus
1	36	50	26	.021	Superior frontal gyrus
2	−2	14	46	.092	Medial frontal gyrus
2	4	22	40	.079	Cingulate gyrus
2	−4	2	60	.038	Medial frontal gyrus
3	12	6	2	.064	Caudate
3	−12	8	4	.046	Caudate
3	12	−14	4	.045	Thalamus
3	−12	−2	12	.029	Caudate
3	−22	6	−2	.025	Lentiform nucleus
3	−12	−10	10	.025	Thalamus
3	−12	−12	2	.024	Thalamus
3	−8	−12	−4	.023	Thalamus
3	−2	−16	6	.023	Thalamus
4	−48	10	26	.059	Inferior frontal gyrus
4	−42	26	24	.039	Middle frontal gyrus
4	−42	0	42	.033	Precentral gyrus
4	−32	2	50	.025	Middle frontal gyrus
5	−32	22	−2	.140	Claustrum
6	−34	−54	46	.065	Inferior parietal lobule
6	−24	−68	46	.028	Precuneus
7	40	−50	46	.056	Inferior parietal lobule
7	34	−60	46	.049	Precuneus
8	−42	−64	−18	.040	Declive

Abbreviations: ALE, Activation Likelihood Estimation; BA, Brodmann Area; MNI, Montreal Neurological Institute.

### 3.5 Behavioral decoding

**FIGURE 6:**
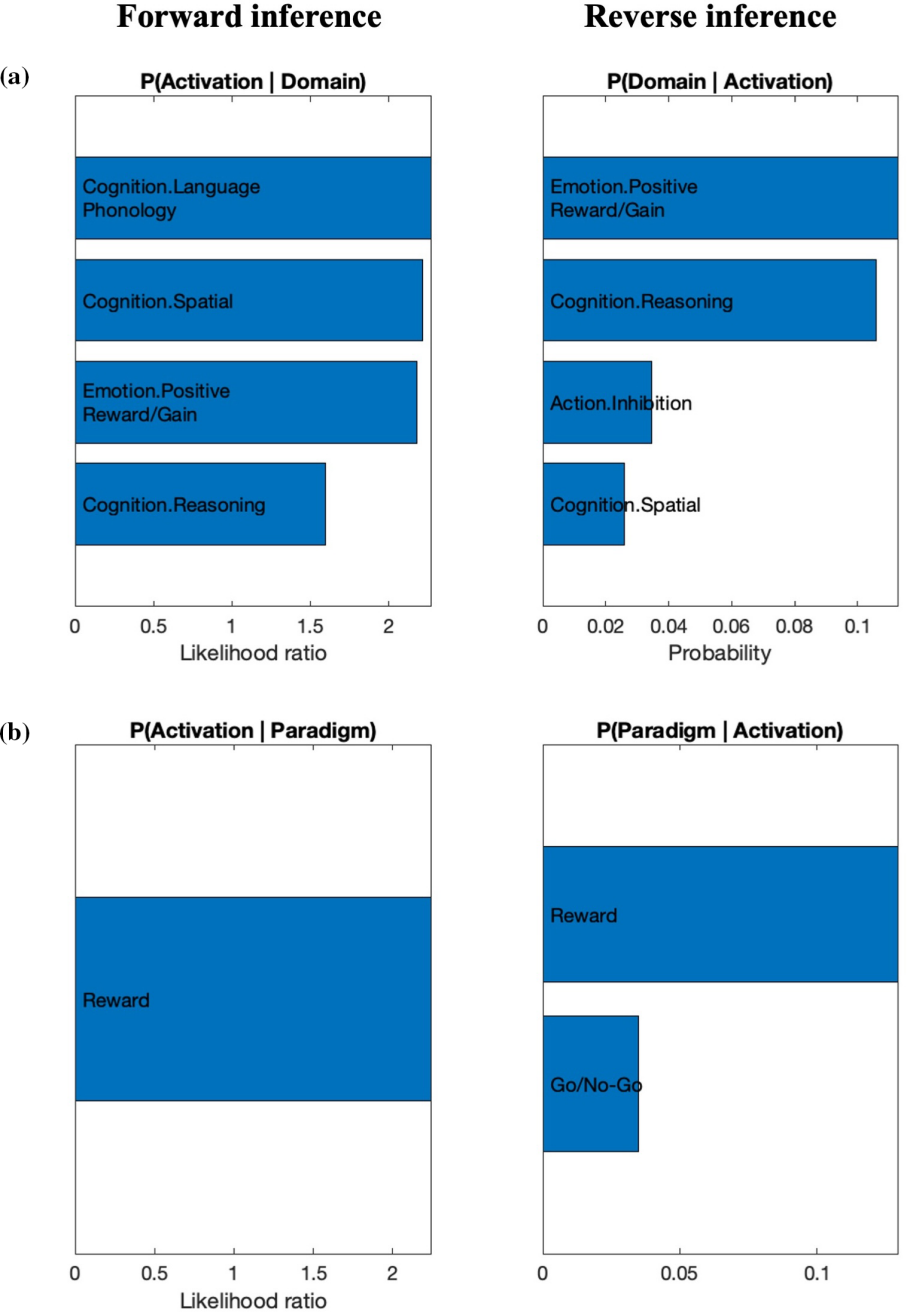
Behavioral decoding of the right anterior insula according to BrainMap. Functional decoding following forward inference is expressed as likelihood ratio (left); functional decoding following reverse inference is expressed as probabilities (right). *A* = Behavioral domains. *B* = Paradigm classes.

Behavioral decoding with the BrainMap database revealed significant associations through both forward and reverse inferences with the BDs of spatial cognition, emotion for positive reward/gain, and cognitive reasoning (Figure 6a). With regards to specific PCs, significant associations across both approaches were found for reward paradigms (Figure 6b).

Neurosynth's association tool identified several behavioral terms associations above the set threshold (*z*‐score ≥ 3), detailed in Table 4. Overall, behavioral terms pertained to reward, effortful processes or cognitive control engagement, memory retrieval or working memory, emotion/mood, and reasoning. In this latter concept, several terms were reminiscent of PP, such as “anticipation,” “decision(s),” “rule,” and “choose.”

**TABLE 4:** Behavioral terms associated with the right anterior insula using Neurosynth's association tool.

Reward‐related terms	
Gain	12.46
Monetary	3.86
Cognitive control‐related terms	
Task	11.64
Task difficulty	7.75
Demands	7.69
Load	6.57
Difficulty	6.48
Tasks	5.94
Cognitive control	5.42
Performance	4.37
Control processes	4.34
Stop	4.32
Interference	4.24
Response times	4.15
Memory retrieval/working memory‐related terms	
Working	6.51
Working memory	6.45
Retrieval	5.64
Memory WM	5.22
Memory	4.99
Memory task	4.75
Maintenance	4.3
Memory retrieval	4.03
Memory load	3.85
WM	3.84
Mood/emotion‐related terms	
Mood	6.46
PTSD	5.08
Reasoning‐related terms	
Anticipation	6.36
Decisions	4.42
Solving	4.28
Rule	4.27
Decision	4.21
Choose	3.97
Other terms	
Phonological	4.72
Correct	4.62
Orthographic	4.59

*Note*: Corresponding *Z*‐scores are reported in the right columns.

### 3.6 Additional analyses

The second minimum conjunction analysis, aimed at formally testing the spatial intersection between the thresholded ALE maps of the cross‐domain prediction phenomena‐independent meta‐analysis and MACM, demonstrated intersection at the level of the right anterior insula (centroid: *x* = 34, *y* = 22, *z* = −2), left anterior insula (centroid: *x* = −33, *y* = 21, *z* = −1), medial frontal gyrus (centroid: *x* = 1, *y* = 18, *z* = 46), right middle frontal gyrus (centroid: *x* = 43, *y* = 32, *z* = 24), right inferior frontal gyrus (centroid: *x* = 47, *y* = 11, *z* = 26), left inferior frontal gyrus (centroid: *x* = −46, *y* = 10, *z* = 27), right inferior parietal gyrus (centroid: *x* = 37, *y* = −54, *z* = 47), and left inferior parietal gyrus (centroid: *x* = −35, *y* = −53, *z* = 47).

Siman‐Tov et al. (2019) results were successfully replicated (Supplementary Information A S5). Domain‐specific SDM analyses yielded consistent results with the ALE approach (Supplementary Information A S6).

## 4 DISCUSSION

The present study employed a meta‐analytic approach to investigate the existence of a domain‐general, large‐scale predictive network encompassing all well‐established cognitive domains, contributing toward a neuroarchitecture conceptualization that goes beyond conventional boundaries of mental terms (Pessoa et al., 2022; Poldrack & Yarkoni, 2016) and supporting PP as a compelling framework for moving toward a more integrated approach to neuroscience. Using the ALE method, the analyses focused on exploring prediction incongruency and prediction congruency, two conditions whose macroscopic metabolic activity is likely reflective of underlying neuronal phenomena of PP. Additionally, PP was investigated in a broader, phenomena‐independent fashion by pooling together datasets on prediction incongruency and prediction congruency. This investigation spanned across all extensively studied cognitive domains (i.e., cognitive control, attention, motor, language, and social cognition), and by aggregating all these cognitive domains into a unified, cross‐domain dimension. Additional analyses were conducted using the MACM method, centering on the right anterior insula as the seed region. This region was identified through the minimum statistic conjunction approach across all cognitive domains under investigation. A further conjunction analysis compared the MACM ALE map with the cross‐domain prediction phenomena‐independent ALE map to formally test for intersections between the two. Finally, to provide a comprehensive perspective on the role of the anterior insula, behavioral decoding was employed to delineate the extensive range of behavioral engagement associated with this multimodal core region.

### 4.1 Toward a domain‐general network: Consistent activations across cognitive tasks and prediction phenomena

**FIGURE 7:**
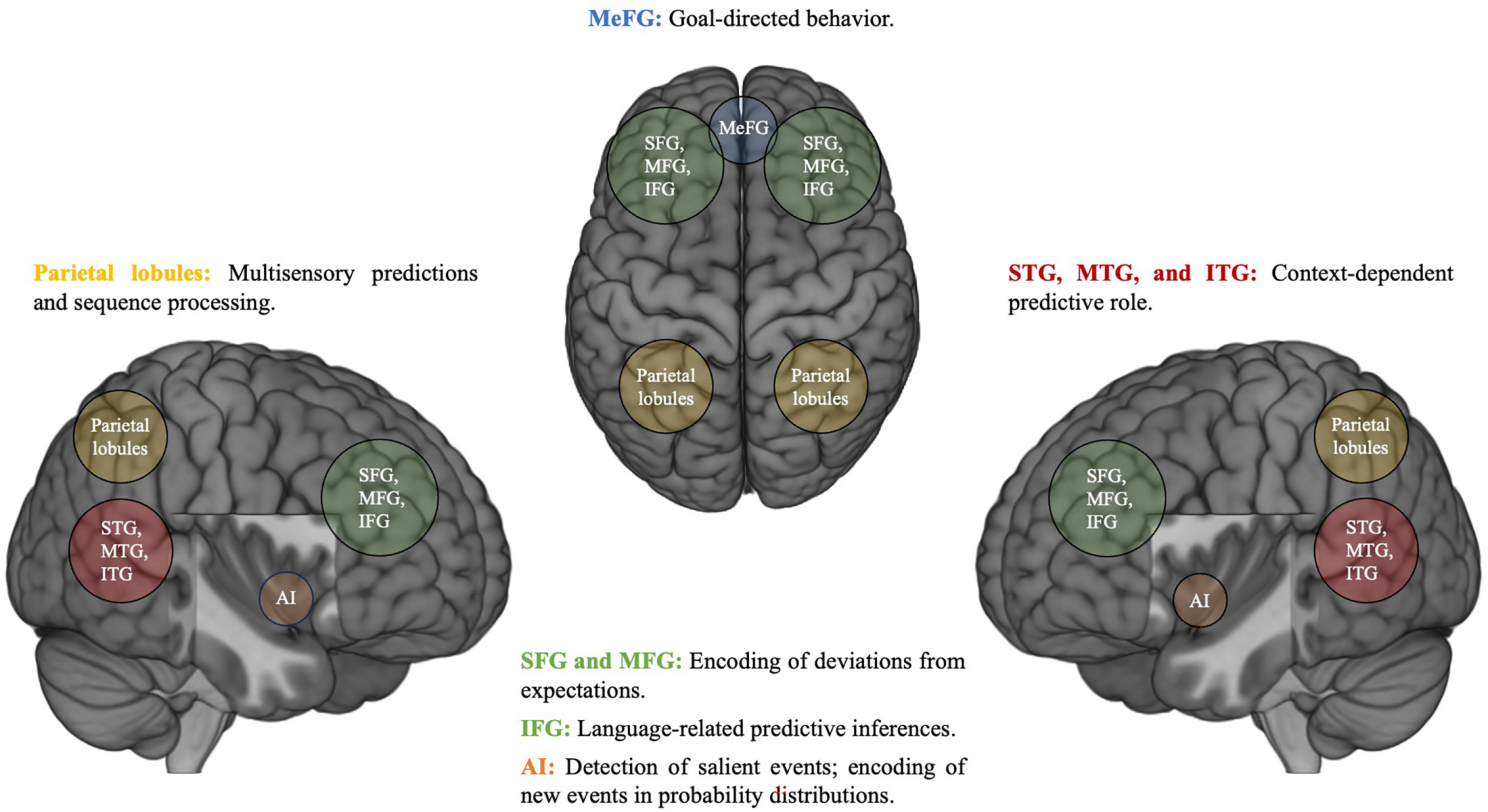
Schematic representation of the Dynamic Prediction Network. A heterarchical structure would allow for a flexible and bidirectional functional relationship between the Dynamic Prediction Network's hubs, within whose regions predictive processing units are likely to operate, depending on the context and environmental demands. This array of regions ultimately integrates context‐ and stimulus‐dependent predictive computations, thereby contributing to the adaptive updating of the brain's models of the inner and external world. AI, anterior insula; IFG, inferior frontal gyrus; ITG, inferior temporal gyrus; MeFG, medial frontal gyrus; MFG, middle frontal gyrus; MTG, middle temporal gyrus; Parietal lobules, superior and inferior parietal lobules; SFG, superior frontal gyrus; STG, superior temporal gyrus.

The results of the present work support the hypothesis that PP relies on a domain‐general, large‐scale network within whose regions PP units are likely to operate, depending on the context and environmental demands. Employing a multi‐faceted approach, combining the ALE meta‐analytic method, MACM, and conjunction analysis, revealed a widely distributed network that appears to support cross‐domain PP (Ficco et al., 2021; Siman‐Tov et al., 2019). This network, which from now on will be referred to as the Dynamic Prediction Network, to emphasize the nature of the between‐hubs interactions arising during predictive computations, encompasses regions within the anterior insula, inferior, middle, and superior frontal gyri, medial frontal gyrus, premotor cortex, inferior and superior parietal lobules, inferior, medial, and superior temporal gyri, temporoparietal junction, caudate, and claustrum (see Figure 7 for a schematic representation). This extensive network, which emerges as a result of the ALE analysis on the cross‐domain prediction phenomena‐independent dataset, finds in the anterior insula one of its core regions, a pivotal node for between‐hub information passing. This (right) region then served as input for the MACM analysis. The remarkable overlap observed between the resultant network from this latter analysis and the cross‐domain prediction phenomena‐independent one also further emphasizes the collective contribution of each hub to predictive computations within a network perspective.

But why should PP rely on a domain‐general, large‐scale network? Numerous studies highlight that neural activity consists of widespread predictions and prediction errors across the brain (e.g., Boyden et al., 2004; Deluca et al., 2014; Johansen et al., 2010; Kok et al., 2017; Rauss et al., 2011; Stefanics et al., 2018, 2019; Zaragoza‐Jimenez et al., 2023). This can be attributed to the continuous generation of predictions and prediction errors by the brain, whether engaged in an experimental task or interacting with the external environment or internal world. These computations encompass various stimulus features, both high and low‐level, spatial, and temporal aspects, as well as somatosensory and visceral information. Ultimately, among other functions, these processes contribute to the generation of conscious experiences (Hohwy & Seth, 2020; Lee et al., 2021; Sklar et al., 2021), whose neural basis spans the neural hierarchy, relying on ongoing predictions and their perturbations by evocative stimuli (e.g., Baker et al., 2022, 2023; Inkster et al., 2022; Stefanics et al., 2018, 2019).

If one's assuming a heterarchical (loosely hierarchical) model (Pessoa, 2019) to interpret the present results, this structure would allow for a flexible and bidirectional functional relationship between brain regions (Lee et al., 2021). In such a model, the context determines whether one brain area is superordinate, subordinate, or equal in rank to another, and direct connections between regions near the bottom and areas near the top can exist without intermediaries, creating a structure well‐suited to support PP architecture (Clark, 2013; Friston, 2008, 2010; Keller & Mrsic‐Flogel, 2018; Lee et al., 2021; Pessoa, 2019; Walsh et al., 2020), and suggesting a parallel organization of psychological processes (Lee et al., 2021). Such an organization would allow for prediction units operating within different sets of brain areas to be recruited depending on the brain state prior to receiving an input, or on the context, in accordance with PP‐consistent degeneracy models (Lupyan & Clark, 2015; Sajid et al., 2020).

In terms of the contribution of each hub to the Dynamic Prediction Network, the involvement of the anterior insula in prediction is crucial and fulfills manifold functions (Corlett et al., 2022). One core function of this hub is the detection of salient events and the initiation of appropriate control signals, establishing its importance as a key region within the Salience Network (SN) (Menon & Uddin, 2010). The anterior insula also plays a critical role in high‐level cognitive control and attentional processes, acting as a “relay” in mediating dynamic interactions between large‐scale brain networks (Nelson et al., 2010). The insula encodes new and unanticipated events in probability density distributions, continuously tested through perceptual reconstruction and sampling (Billeke et al., 2020). This is also demonstrated by direct electrophysiological evidence of its leading role within the Error‐Monitoring Network, rapidly detecting and conveying error signals to the dorsomedial prefrontal cortex (Bastin et al., 2016). In terms of functional connectivity, the insula is part of a large bilateral predictive network that resembles networks involved in task‐driven attention and execution (Barrós‐Loscertales, 2018), as also highlighted by MACM results. Additionally, behavioral decoding here emphasizes that the right anterior insula is involved in coordinating brain circuits related to reward‐based learning, relaying feedback information signals to the medial prefrontal cortex (Rousseau et al., 2021). Overall, the anterior insula, implicated at a cross‐domain, multimodal level in both prediction congruency and incongruency, constitutes the major hub of the Dynamic Prediction Network.

The contribution of the frontal gyri to predictive processes is mainly related to encoding stimuli in terms of deviations from expectation, adjusting neural dynamics according to the average time of an expected stimulus, and processing information predictively based on temporal events (Ficco et al., 2021; Meirhaeghe et al., 2021). Multiple pieces of evidence support the idea that the left inferior frontal gyrus is responsible for constructing predictive inferences (Jin et al., 2009), mainly related to language processing. For instance, Söderström et al. (2017) found that the left inferior frontal gyrus is involved in resolving competition between activated word endings to achieve automatic and predictive processes of syntax (it is noteworthy to mention that the role of the inferior frontal gyrus in prediction during language comprehension is still debated, see Maran et al., 2022). Additionally, the frontal lobes, including the lateral and medial prefrontal cortex, are implicated in anticipating prediction errors and performing goal‐directed behavior, providing a unified account of the prefrontal cortex functioning (Alexander & Brown, 2018).

The premotor cortex and parietal lobules' role in PP revolves around multisensory predictions and sequence processing (D'Mello & Rozenkrantz, 2020; Downing, 2013; Gastaldon et al., 2023; Meirhaeghe et al., 2021). Moreover, the premotor cortex is implicated in action perception and motor control, both inherently involved in prediction (Ficco et al., 2021; Siman‐Tov et al., 2019). On the other hand, the parietal lobules are involved in action understanding and imitation (Miller & Clark, 2018).

As highlighted in a recent systematic review (Masina et al., 2022), the right temporoparietal junction serves as a central hub not only within the Ventral Attention Network and the Default Mode Network (DMN), but also in a broader hierarchical prediction network that plays a role in determining the internal model of the task context (Geng & Vossel, 2013) and attentional processes (Wilterson et al., 2021). The discussion posits that the role of the right temporoparietal junction in PP is context‐dependent, not strictly tied to a particular prediction network. Instead, its specific function is shaped by the network engaged in the ongoing task or activity, with the context determining its coupling with other regions and aligning with task‐specific networks (Masina et al., 2022). This makes the right temporoparietal junction a pivotal hub for PP, facilitating maximum flexibility in integrating various cognitive processes and engaging different brain regions (Masina et al., 2022).

The cerebellum constitutes an expected site for convergence when analyzing prediction‐related data, particularly prediction violations (Boyden et al., 2004; Deluca et al., 2014). However, convergence in this area failed to reach statistical significance, except for the declive, the interpretation of which remains challenging. Ficco et al. (2021) also reported this unexpected (null) finding and offered a plausible explanation. The authors suggest that the reason behind a few meta‐analyses detecting convergence in this area (Siman‐Tov et al., 2019) may be attributed to technical challenges associated with detecting the BOLD signal from the cerebellum, especially in experiments targeting climbing fibers. Moreover, certain experimental paradigms can lead to rapid habituation in the cerebellum, resulting in lower neural responses (Ficco et al., 2021).

Placing these findings into context, the Dynamic Prediction Network encompasses a wide array of pivotal hubs shared with established networks, including the DMN, the Executive Control Network, the SN, the Dorsal and Ventral Attention Networks, and the Error‐Monitoring Network. The (qualitatively) observed overlap with these networks offers intriguing insights into the interplay between PP and cognitive processes. For instance, the convergence of the SN with the Dynamic Prediction Network aligns with the SN's role in directing attention toward unexpected or significant stimuli. Additionally, the co‐localization of the Dorsal Attention Networks within the Dynamic Prediction Network speaks to its involvement in optimizing prediction refinements. The Executive Control Network likely facilitates the adaptive updating of predictions based on contextual demands. Lastly, the presence of DMN regions within the Dynamic Prediction Network suggests that internally focused brain processes also engage in predictive computations.

The present results build upon and expand previous literature (Corlett et al., 2022; Ficco et al., 2021; Siman‐Tov et al., 2019). By including a high number of relevant experiments and thus increasing signal precision, the present study reiterates the key role of already identified prediction hubs, such as the insula, while providing a broader picture of where in the brain prediction units may operate based on contextual circumstances. This holistic perspective highlights the brain's ability to seamlessly integrate predictive computations across cognitive domains, leading to a deeper understanding of its dynamic nature. It is plausible to assume that the Dynamic Prediction Network hubs, depending on the context and task/environment demands, engage in generating, evaluating, and refining predictions to adaptively update the brain's models of both the inner and external world, all while integrating with well‐established cognitive networks.

### 4.2 To each its own: Consistent activations within cognitive tasks and prediction phenomena

Delving into the results related to predictive processes within the domains of cognitive control, attention, language, motor, and social cognition, a delicate balance of context‐dependent flexibility and generalization was anticipated. It is well‐established that the human brain exhibits remarkable plasticity, allowing for both specialized processing within certain regions and distributed networks that facilitate cross‐domain interactions (Herbet & Duffau, 2020; Masina et al., 2022; Sherwood & Gómez‐Robles, 2017). In this regard, expectations were twofold: the emergence of areas demonstrating domain‐specific and prediction phenomena‐specific (i.e., subtending either congruency or incongruency) engagement, and the identification of areas that might exhibit broader involvement, contributing to the manifold entanglement of cognitive functions. The study's findings regarding domain‐specific prediction phenomena highlight a prominent pattern: results pertaining to prediction incongruency appear to rely on a defined network across cognitive domains, prominently involving the inferior frontal gyrus, insula, claustrum, parietal lobules, and temporal gyri. Conversely, prediction congruency results exhibited less overall activation and slightly more variability across cognitive domains. These results highlight the brain's ability to flexibly use its resources to maintain efficient processing and support various cognitive tasks (Herbet & Duffau, 2020).

In the context of cognitive control, the main areas showing consistent activation for the prediction congruency analysis were the right superior and middle frontal gyri. On the other hand, the meta‐analysis on prediction incongruency highlighted the involvement of a more extended set of brain areas. The emergence of a cluster encompassing both the superior and middle frontal gyri suggests their role in prediction‐related computations, irrespective of specific phenomena, whereas the inferior frontal gyrus exhibited specificity for detecting prediction deviations in tasks related to cognitive control (Sherman et al., 2016). Similarly, the insula and claustrum, while generally playing more of a prediction phenomena‐independent role, displayed a potential specialization for signaling prediction incongruencies within this cognitive domain. Besides the previously discussed role of insula, the claustrum is believed to contribute to PP via its connections to structures involved in attention and salience processing, such as the anterior cingulate cortex and insular cortex (Benarroch, 2021).

Disentangling the role of areas showing consistent activation in the context of attention is a complex task, as the ongoing discussion about the influence of attention on PP remains active. The left cingulate gyrus, involved in conflict‐related task performance, selective attention, and cognitive flexibility (Merkley et al., 2013; Turak et al., 2002), may play a role in establishing and updating event models that can predictively guide perception, learning, and action control (Stawarczyk et al., 2021). On the other hand, an overall more distributed, bilateral fronto‐parieto‐temporal network is responsible for increased attentive selectivity to mismatch information and optimizing the expected precision of predictions (Dreneva et al., 2021).

In the motor domain, prediction congruency specificity is observed in the left supramarginal gyrus, a region known to play a role in motor functions, particularly the representation of meaningful actions (praxis) (Króliczak et al., 2016) and planning and execution of familiar actions (Guidali et al., 2019). The manifold role of the left supramarginal gyrus aligns seamlessly with what one would expect from a prediction hub underlying motor action, encompassing the integration of conceptual knowledge into purposeful movements (Hartwigsen et al., 2012; Potok et al., 2019). On the other hand, prediction incongruency correlates mainly rely on activity within the insula and claustrum.

Regarding the language domain, both prediction congruency and incongruency correlates exhibit clear left lateralization. Within this cognitive domain, the left inferior frontal gyrus, insula, and middle temporal gyrus play a prediction phenomena‐independent role. The inferior temporal gyrus is well‐known for its involvement in language processing (Medaglia et al., 2021; Rivas‐Fernández et al., 2021) and prediction (Iijima & Sakai, 2014; Söderström et al., 2017; Strijkers et al., 2019; but see Maran et al., 2022 for a different point of view). Concerning the left middle temporal gyrus, it has been shown that predictability impacts activity in this region, highlighting its role as a key contributor to the effects of predictability on comprehension (Lau & Namyst, 2019). The present results align with the left middle temporal gyrus being implicated in various aspects of PP and conceptual representation. The insula cortex exhibited discernable specificity towards predictive phenomena in language processing, manifesting through lateralization. Specifically, the left insula assumes a central role in signaling prediction incongruencies, likely substantiating the identification and processing of deviations from expected patterns (Ficco et al., 2021). In contrast, the right insula emerges as a key region in the encoding of predictive linguistic elements, emphasizing its role in the generation of prediction codes (Billeke et al., 2020) related to linguistic constructs.

Lastly, whereas prediction incongruency pertaining to social cognition showed consistent activation of the right claustrum and inferior frontal gyrus, consistent activation was found in the right caudate nucleus regarding prediction congruency. This region plays an important role in social cognition (Myznikov et al., 2021), as also indicated by findings showing that high social intelligence scores positively correlate with larger gray matter volumes of the bilateral caudate (Sokolov, 2018).

## 5 LIMITATIONS

The current study, while providing valuable insights into the PP framework, is not devoid of potential limitations that warrant consideration, encompassing both theoretical constraints within the cognitive neuroscience field and methodological challenges.

First, our reliance on classifying data according to cognitive domains is confronted by the absence of a widely accepted and dominant framework for their definition. The field's lack of consensus, as highlighted by Muthukrishna and Henrich (2019), introduces caution regarding the generalizability of our findings. The predefined segregation of cognitive domains, designed to facilitate analysis, introduces a limitation due to inherent overlaps between these domains and to the lack of coverage of the complete spectrum of cognitive functions. Our a‐priori categorization may not fully capture the intricate interconnections and shared neural substrates across different cognitive domains. Nevertheless, exploring overall results across domains offers valuable insights into potential cross‐domain relationships. Second, a disparity in clarity and comparability emerges between the study of prediction incongruency and prediction congruency. Events like prediction violations, marked by surprise and mismatch, provide a more distinct and well‐defined focus. This clarity not only influences the inclusion of studies in our meta‐analysis but also underscores a broader limitation in the existing literature. The challenge lies in the variability and complexity associated with studying the formation and updating of predictions.

As our work focused on identifying brain areas engaged during prediction congruency and incongruency conditions, providing spatial information, we lack data on the millisecond‐level processes involved. This temporal information is undoubtedly important in understanding predictive processes and could be valuable for future efforts aiming at integrating the present findings with EEG/MEG evidence.

These limitations collectively highlight avenues for future research to grapple with the inherent challenges in the study of PP, and the present results could be informative in guiding such research by providing evidence for the contribution of a wide array of hubs from a network perspective.

## 6 CONCLUSIONS

This study employed the ALE meta‐analytic approach, along with methods such as MACM, conjunction analysis, and behavioral decoding, to investigate the neural implementation of PP across well‐established cognitive domains. The aim was to test the hypothesis that PP relies on a domain‐general, large‐scale network within whose regions PP units are likely to operate, depending on the context and environmental demands, contributing toward a neuroarchitecture conceptualization that goes beyond conventional boundaries of mental terms (Pessoa et al., 2022; Poldrack & Yarkoni, 2016), and supporting PP as a unified account toward a more integrated approach to neuroscience. Consistent activations observed across diverse cognitive domains and prediction conditions (i.e., prediction congruency and incongruency) underscore the role of several key hubs (e.g., insula, frontal gyri, temporal gyri, parietal lobules, claustrum) within the Dynamic Prediction Network. The results of the present work thus support the hypothesis that PP relies on a domain‐general, large‐scale network. In fact, if framed within a heterarchical organization that emphasizes the nature of the between‐hubs interactions, the wide array of regions within the Dynamic Prediction Network seamlessly integrate context‐ and stimulus‐dependent predictive computations, contributing to the adaptive updating of the brain's models of the inner and external world. Such an organization would allow for prediction units operating within different sets of brain areas to be recruited depending on the context and environmental demands.

Insights into the neural underpinnings of PP hold promise for neurologic and psychiatric applications, providing a foundation for targeted interventions in conditions characterized by aberrant predictive processes (Feeney et al., 2017; Manjaly et al., 2019; Migeot et al., 2022; Owens et al., 2018; Stephan et al., 2006).

Lastly, building on the current findings, several avenues for future research emerge, including the exploration of temporal dynamics of predictions, understanding inter‐individual differences, and employing multimodal approaches. These avenues may further refine our understanding of the neural implementation of PP, paving the way for innovative applications in clinical and neuroscientific domains.

## AUTHOR CONTRIBUTIONS

CC, RP, FM, GA, and CS conceived the study and wrote the first draft of the manuscript. CC, RP, FM, SL, and SG collected the data. CC and CF analyzed the data. All authors contributed to the interpretation of the results, writing, and editing of the paper.

## CONFLICT OF INTEREST STATEMENT

The authors declare no competing interests.

## Supporting information


Data S1.

Data S2.

Data S3.

Data S4.

## Data Availability

The data that support the findings of this study are openly available in OSF at https://osf.io/cz5hn/, reference number osf.io/529qg.
