# Supplementary material for: Comprehensive investigation of predictive processing: A cross‐ and within‐cognitive domains fMRI meta‐analytic approach
Source: Hum Brain Mapp. 2024 Aug 21;45(12):e26817. doi: 10.1002/hbm.26817 (PMC11339134; doi:10.1002/hbm.26817)
Supplement: Supplementary file 1 — Data S1. [file HBM-45-e26817-s004.pdf]

**Supplementary Information A**

# Comprehensive Investigation of Predictive Processing: A Cross- and Within-Cognitive Domains fMRI Meta-Analytic Approach

Cristiano Costa[1], Rachele Pezzetta[2*], Fabio Masina[2], Sara Lago[2], Simone Gastaldon[1,3], Camilla Frangi[4], Sarah Genon[5,6], Giorgio Arcara[2], Cristina Scarpazza[2,4]

[1]Padova Neuroscience Center, Padua, Veneto, Italy
[2]IRCCS Ospedale San Camillo, Venice, Veneto, Italy
[3]Università degli Studi di Padova, Dipartimento di Psicologia dello Sviluppo e della Socializzazione, Padua, Veneto, Italy
[4]Università degli Studi di Padova, Dipartimento di Psicologia Generale, Padua, Veneto, Italy
[5]Institute for Systems Neuroscience, Heinrich Heine University Düsseldorf, Düsseldorf, Germany
[6]Institute of Neuroscience and Medicine, Brain & Behaviour (INM-7), Research Centre Jülich, Jülich, Germany

[*]**Corresponding author:** Rachele Pezzetta (rachele.pezzetta@hsancamillo.it)

**Supplementary Information A S1:** Complete search strings
**Supplementary Information A S2:** Domain-specific ALE results divided for congruency and incongruency
**Supplementary Information A Figure S1:** Graphical representation of domain-specific ALE results divided for congruency and incongruency
**Supplementary Information A S3:** Contrast analysis between cross-domain prediction incongruency and cross-domain prediction congruency results
**Supplementary Information A S4:** Number of included experiments for each dataset
**Supplementary Information A S5:** Siman-Tov et al., 2019 replication
**Supplementary Information A S6:** Seed-based d Mapping Meta-Analyses
**Supplementary Information A S7:** References of studies included in the meta-analyses

**Supplementary Information A S1:** Complete search strings

For each database searched (i.e., Pubmed, Embase, PsychINFO), the general research string is provided, while domain-specific strings are reported in tables. These tables also present the number of papers found for each domain.

**Pubmed**

(((neuroimaging [Title/Abstract] OR fMRI[Title/Abstract] OR functional MRI [Title/Abstract] OR magnetic resonance imaging[Title/Abstract] OR PET[Title/Abstract]) NOT (DTI[Title/Abstract] OR Diffusion tensor Imaging[Title/Abstract] OR machine learning[Title/Abstract] OR structural[Title/Abstract] OR tDCS[Title/Abstract] OR TMS[Title/Abstract] OR EEG[Title/Abstract] OR intracranial[Title/Abstract]))

AND

(prediction[Title/Abstract] OR expectancy[Title/Abstract] OR expectation[Title/Abstract] OR anticipation[Title/Abstract] OR unexpected[Title/Abstract] OR surpris*[Title/Abstract] OR prediction error[Title/Abstract] OR incongruent[Title/Abstract] OR irregular[Title/Abstract] OR violat*[Title/Abstract] OR mismatch[Title/Abstract] OR anticipat*[Title/Abstract])

AND

**INSERT HERE THE DOMAIN-SPECIFIC SEARCH STRING**

NOT (disease[Title/Abstract] OR disorder[Title/Abstract] OR pathology[Title/Abstract] OR psychiat*[Title/Abstract] OR stroke[Title/Abstract] OR neurologic*[Title/Abstract] OR Alzheimer[Title/Abstract] OR Parkinson[Title/Abstract] OR depression[Title/Abstract] OR schizophrenia[Title/Abstract] OR dementia[Title/Abstract] OR neglect[Title/Abstract] OR drug*[Title/Abstract] OR brain injury[Title/Abstract] OR surgery[Title/Abstract] OR surgical[Title/Abstract] OR damag*[Title/Abstract] OR animal*[Title/Abstract] OR infants[Title/Abstract] OR adolescents[Title/Abstract] OR older[Title/Abstract] OR elderly[Title/Abstract] OR child*[Title/Abstract] OR phantom[Title/Abstract] OR rats[Title/Abstract] OR aphasi*[Title/Abstract] OR sign[Title/Abstract] OR adhd[Title/Abstract] OR deficit*[Title/Abstract] OR patient*[Title/Abstract] OR single-case[Title/Abstract] OR rodent*[Title/Abstract] OR review[Publication Type] OR meta-analysis[Publication Type] OR developmental[Title/Abstract] OR infants[Title/Abstract] OR surprisingly[Title/Abstract] OR unexpected results[Title/Abstract] OR unexpectedly[Title/Abstract] OR treatment[Title/Abstract] OR training[Title/Abstract] OR longitudinal[Title/Abstract])

| Domain | Research string | Papers obtained |
|---|---|---|
| Cognitive Control | (executive function*[Title/Abstract] OR cognitive flexibility[Title/Abstract] OR perseverat*[Title/Abstract] OR cognitive control[Title/Abstract] OR error-monitoring[Title/Abstract] OR error monitoring[Title/Abstract] OR error detection[Title/Abstract] OR cognitive conflict[Title/Abstract]) | 200 results |

| | | |
|---|---|---|
| Attention | (spatial attention[Title/Abstract] OR external attention[Title/Abstract] OR selective attention[Title/Abstract] OR sustained attention[Title/Abstract] OR focused attention[Title/Abstract] OR alternated attention[Title/Abstract] or divided attention[Title/Abstract] OR visual attention[Title/Abstract] OR attention[Title/Abstract]) | 373 results |
| Language | (language[Title/Abstract] OR linguistic[Title/Abstract] OR semantic*[Title/Abstract] OR syntactic[Title/Abstract] OR phonological[Title/Abstract] OR pragmatic*[Title/Abstract]) | 410 results |
| Motor | (motor[Title/Abstract] OR motor planning[Title/Abstract] OR movement[Title/Abstract] OR kinematic*[Title/Abstract]) | 436 results |
| Social Cognition | (social cognition[Title/Abstract] OR theory of mind[Title/Abstract] OR mentalizing[Title/Abstract] OR mindreading[Title/Abstract] OR mind-reading[Title/Abstract] OR mind reading[Title/Abstract] OR ToM[Title/Abstract] OR social learning[Title/Abstract]) | 73 results |
| Memory | (prospective memory[Title/Abstract] OR episodic memory[Title/Abstract] OR semantic memory[Title/Abstract] OR procedural memory[Title/Abstract] OR long-term memory[Title/Abstract] OR working memory[Title/Abstract] OR short-term memory[Title/Abstract]) | 192 results |
| Music | (music*[Title/Abstract] OR harmonic[Title/Abstract] OR melodic[Title/Abstract] OR rhythmic[Title/Abstract]) | 40 results |
| Pain | pain[Title/Abstract] | 143 results |

**Embase**

(neuroimaging OR fMRI OR functional MRI OR magnetic resonance imaging OR PET) NOT (DTI OR Diffusion tensor Imaging OR machine learning OR structural OR tDCS OR TMS OR EEG OR intracranial)

AND

(prediction OR expectancy OR expectation OR anticipation OR unexpected OR surpris* OR prediction error OR incongruent OR irregular OR violat* OR mismatch OR anticipat*)

AND

**INSERT HERE THE DOMAIN-SPECIFIC SEARCH STRING**

NOT (disease OR disorder OR pathology OR psychiat* OR stroke OR neurologic*OR Alzheimer OR Parkinson OR depression OR schizophrenia OR dementia OR neglect OR drug* OR brain injury OR surgery OR surgical OR damag* OR animal* OR infants OR adolescents OR older OR elderly OR child* OR phantom OR rats OR aphasi*OR sign OR adhd OR deficit OR patient* OR single-case OR rodent* OR review OR meta-analysis OR developmental OR infants OR surprisingly OR unexpected results OR unexpectedly OR treatment OR training OR longitudinal)

| Domain | Research string | Papers obtained |
|---|---|---|
| Cognitive Control | (executive function* OR cognitive flexibility OR perseverat* OR cognitive control OR error-monitoring OR error monitoring OR error detection OR cognitive conflict) | 124 results |
| Attention | (attention) | 267 results |
| Language | (language OR semantic* OR syntactic OR pragmatic*) | 165 results |
| Motor | (motor planning OR movement OR kinematic*) | 278 results |
| Social Cognition | (social cognition OR theory of mind OR mentalizing OR mindreading OR mind-reading OR mind reading OR ToM OR social learning) | 115 results |
| Memory | (prospective memory OR episodic memory OR semantic memory OR procedural memory OR long-term memory OR working memory OR short-term memory) | 69 results |
| Music | (music* OR harmonic OR melodic OR rhythmic) | 49 results |
| Pain | (pain) | 150 results |

**PsychInfo**
((ab(neuroimaging) OR ab(fMRI) OR ab(functional MRI) OR ab(magnetic resonance imaging) OR ab(PET)) NOT (ab(DTI) OR ab(Diffusion tensor Imaging) OR ab(machine learning) OR ab(structural) OR ab(tDCS) OR ab(TMS) OR ab(EEG) OR ab(intracranial)))
AND
(ab(prediction) OR ab(expectancy) OR ab(expectation) OR ab(anticipation) OR ab(unexpected) OR ab(surpris*) OR ab(prediction error) OR ab(incongruent) OR ab(irregular) OR ab(violat*) OR ab(mismatch) OR ab(anticipat*))
AND
**INSERT HERE THE DOMAIN-SPECIFIC SEARCH STRING**
NOT (ab(disease) OR ab(disorder) OR ab(pathology) OR ab(psychiat*) OR ab(stroke) OR ab(neurologic*) OR ab(Alzheimer) OR ab(Parkinson) OR ab(depression) OR ab(schizophrenia) OR ab(dementia) OR ab(neglect) OR ab(drug*) OR ab(brain injury) OR ab(surgery) OR ab(surgical) OR ab(damag*) OR ab(animal*) OR ab(infants) OR ab(adolescents) OR ab(older) OR ab(elderly) OR ab(child*) OR ab(phantom) OR ab(rats) OR ab(aphasi*) OR ab(sign) OR ab(adhd) OR ab(deficit) OR ab(patient*) OR ab(single-case) OR ab(rodent*) OR ab(review) OR ab(meta-analysis) OR ab(developmental) OR ab(infants)OR ab(surprisingly) OR ab(unexpected results) OR ab(unexpectedly) OR ab(treatment) OR ab(training) OR ab(longitudinal))

| Domain | Research string | Papers obtained |
|---|---|---|
| Cognitive Control | (ab(executive function*) OR ab(cognitive flexibility) OR ab(@perseverat*) OR ab(cognitive control) OR ab(error monitoring) OR ab(error-monitoring) OR ab(error detection) OR ab(cognitive conflict)) | 271 results |
| Attention | (ab(attention)) | 338 results |
| Language | (ab(language) OR ab(semantic*) OR ab(syntactic) OR ab(pragmatic*)) | 316 results |
| Motor | (ab(motor) OR ab(motor planning) OR ab(movement) OR ab(kinematic*)) | 437 results |
| Social Cognition | (ab(social cognition) OR ab(theory of mind) OR ab(mentalizing) OR ab(mindreading) OR ab(mind-reading) OR ab(mind reading) OR ab(ToM) OR ab(social learning)) | 98 results |
| Memory | (ab(prospective memory) OR ab(episodic memory) OR ab(semantic memory) OR ab(procedural memory) OR ab(long-term memory) OR ab(working memory) OR ab(short-term memory))) | 246 results |
| Music | (ab(music*) OR ab(harmonic) OR ab(melodic) OR ab(rhythmic)) | 54 results |
| Pain | (ab(pain)) | 29 results |

**Supplementary Information A S2:** Domain-specific ALE results divided for congruency and incongruency

| Congruency | | | | Incongruency | | | |
|---|---|---|---|---|---|---|---|
| **Cluster** | **MNI Coordinates** | **Peak ALE p-value** | **Region** | **Cluster** | **MNI Coordinates** | **Peak ALE p-value** | **Region** |
| **ATTENTION** | | | | | | | |
| 1 | -6 10 48 | 0.023 | Cingulate gyrus | 1 | -2 14 50 | 0.052 | Superior frontal gyrus |
| 1 | 6 14 50 | 0.020 | Superior frontal gyrus | 2 | 48 14 28 | 0.038 | Inferior frontal gyrus |
| 2 | -42 -34 20 | 0.021 | Superior temporal gyrus | 2 | 48 6 34 | 0.036 | Precentral gyrus |
| 2 | -52 -20 20 | 0.014 | Insula | 2 | 38 8 28 | 0.023 | Precentral gyrus |
| 2 | -54 -30 24 | 0.014 | Inferior parietal lobule | 2 | 56 22 32 | 0.021 | Middle frontal gyrus |
| 3 | 42 -4 50 | 0.016 | Precentral gyrus | 3 | 30 -66 44 | 0.029 | Precuneus |
| 3 | 38 -8 50 | 0.016 | Precentral gyrus | 3 | 36 -60 46 | 0.028 | Precuneus |
| 3 | 34 -10 56 | 0.015 | Precentral gyrus | 3 | 42 -46 46 | 0.025 | Inferior parietal lobule |
| | | | | 3 | 32 -64 36 | 0.019 | Precuneus |
| | | | | 4 | 38 20 0 | 0.037 | Insula |
| | | | | 5 | -34 22 -4 | 0.035 | Insula |
| | | | | 6 | 32 2 58 | 0.037 | Sub-gyral |
| | | | | 7 | -36 -46 42 | 0.023 | Inferior parietal lobule |
| | | | | 7 | -42 -42 48 | 0.021 | Inferior parietal lobule |
| | | | | 7 | -26 -56 48 | 0.017 | Superior parietal lobule |
| | | | | 8 | -28 0 56 | 0.027 | Middle frontal gyrus |
| | | | | 8 | -30 -8 48 | 0.017 | Middle frontal gyrus |
| | | | | 9 | 64 -34 6 | 0.024 | Superior temporal gyrus |
| | | | | 9 | 56 -44 16 | 0.023 | Superior temporal gyrus |

| | | | | | | | |
|---|---|---|---|---|---|---|---|
| | | | | 10 | -58 -26 10 | 0.022 | Superior temporal gyrus |
| | | | | 10 | -62 -38 14 | 0.020 | Superior temporal gyrus |
| **COGNITIVE CONTROL** | | | | | | | |
| 1 | 32 56 -4 | 0.023 | Middle frontal gyrus | 1 | -44 12 28 | 0.026 | Inferior frontal gyrus |
| 2 | 56 -44 50 | 0.033 | Inferior parietal lobule | 1 | -48 18 10 | 0.024 | Inferior frontal gyrus |
| 3 | 4 20 50 | 0.023 | Superior frontal gyrus | 1 | -42 24 22 | 0.021 | Middle frontal gyrus |
| 4 | -24 -70 -32 | 0.028 | Pyramis | 1 | -54 22 22 | 0.017 | Inferior frontal gyrus |
| | | | | 1 | -48 14 42 | 0.016 | Middle frontal gyrus |
| | | | | 2 | -2 14 50 | 0.044 | Superior frontal gyrus |
| | | | | 2 | -6 34 44 | 0.014 | Medial frontal gyrus |
| | | | | 2 | -8 30 40 | 0.014 | Cingulate gyrus |
| | | | | 3 | 34 22 2 | 0.028 | Claustrum |
| | | | | 3 | 48 32 8 | 0.021 | Inferior frontal gyrus |
| | | | | 3 | 38 22 18 | 0.020 | *No gray matter found* |
| | | | | 3 | 50 26 20 | 0.017 | Middle frontal gyrus |
| | | | | 3 | 48 24 -2 | 0.016 | Inferior frontal gyrus |
| | | | | 4 | -32 22 -2 | 0.030 | Claustrum |
| | | | | 4 | -34 20 6 | 0.027 | Insula |
| | | | | 5 | 44 12 38 | 0.021 | Precentral gyrus |
| | | | | 5 | 42 14 26 | 0.019 | Inferior frontal gyrus |
| | | | | 5 | 50 18 28 | 0.015 | Inferior frontal gyrus |
| | | | | 6 | -44 -42 44 | 0.026 | Inferior parietal lobule |

| | | | | | | | |
|---|---|---|---|---|---|---|---|
| | | | | 6 | -38 -48 40 | 0.016 | Inferior parietal lobule |
| | | | | 7 | -28 0 48 | 0.019 | Middle frontal gyrus |
| | | | | 8 | -34 -62 46 | 0.020 | Inferior parietal lobule |
| | | | | 8 | -30 -70 38 | 0.017 | Precuneus |
| **LANGUAGE** | | | | | | | |
| 1 | -48 34 -6 | 0.029 | Inferior frontal gyrus | 1 | -42 16 20 | 0.028 | Middle frontal gyrus |
| 1 | -48 40 8 | 0.028 | Inferior frontal gyrus | 1 | -54 18 16 | 0.026 | Inferior frontal gyrus |
| 2 | -56 -38 4 | 0.022 | Middle temporal gyrus | 1 | -50 32 10 | 0.021 | Inferior frontal gyrus |
| 2 | -54 -38 -4 | 0.017 | Middle temporal gyrus | 1 | -42 6 30 | 0.021 | Precentral gyrus |
| 2 | -60 -50 10 | 0.014 | Middle temporal gyrus | 1 | -44 8 38 | 0.018 | Middle frontal gyrus |
| 3 | -44 -56 46 | 0.022 | Inferior parietal lobule | 1 | -44 20 34 | 0.017 | Precentral gyrus |
| 4 | -48 -48 -14 | 0.020 | Fusiform gyrus | 1 | -40 18 6 | 0.016 | Insula |
| 5 | -56 22 16 | 0.021 | Inferior frontal gyrus | 1 | -52 30 20 | 0.013 | Middle frontal gyrus |
| 5 | -46 18 20 | 0.017 | Inferior frontal gyrus | 2 | -56 -42 6 | 0.027 | Middle temporal gyrus |
| 6 | 32 26 -4 | 0.019 | Insula | 2 | -58 -46 4 | 0.027 | Middle temporal gyrus |
| 6 | 32 22 -6 | 0.019 | Claustrum | 2 | -60 -32 0 | 0.022 | Middle temporal gyrus |
| | | | | 2 | -66 -38 12 | 0.018 | Superior temporal gyrus |
| | | | | 3 | -32 24 -2 | 0.029 | Insula |
| | | | | 3 | -44 28 -4 | 0.016 | Inferior frontal gyrus |
| | | | | 4 | 42 32 -8 | 0.024 | Inferior frontal gyrus |
| | | | | 4 | 52 34 -2 | 0.023 | Inferior frontal gyrus |

| | | | | | | | |
|---|---|---|---|---|---|---|---|
| | | | | 4 | 52 36 6 | 0.021 | Inferior frontal gyrus |
| | | | | 4 | 50 24 12 | 0.016 | Inferior frontal gyrus |
| | | | | 5 | -4 18 48 | 0.026 | Superior frontal gyrus |
| | | | | 5 | 6 18 44 | 0.025 | Medial frontal gyrus |
| | | | | 5 | 10 30 40 | 0.015 | Cingulate gyrus |
| **MOTOR** | | | | | | | |
| 1 | -52 -52 32 | 0.017 | Supramarginal gyrus | 1 | 36 18 0 | 0.026 | Claustrum |
| 1 | -58 -46 40 | 0.017 | Supramarginal gyrus | 1 | 34 22 8 | 0.016 | Insula |
| | | | | 2 | -32 20 -6 | 0.023 | Claustrum |
| | | | | 2 | -42 14 2 | 0.020 | Insula |
| | | | | 3 | 6 12 50 | 0.017 | Superior frontal gyrus |
| | | | | 3 | 6 24 42 | 0.016 | Cingulate gyrus |
| | | | | 3 | -2 28 42 | 0.015 | Medial frontal gyrus |
| **SOCIAL COGNITION** | | | | | | | |
| 1 | 12 22 -12 | 0.020 | Caudate | 1 | 34 20 -4 | 0.027 | Claustrum |
| | | | | 1 | 42 20 -12 | 0.018 | Inferior frontal gyrus |

**Supplementary Information A Figure S1:** Graphical representation of domain-specific ALE results divided for congruency and incongruency

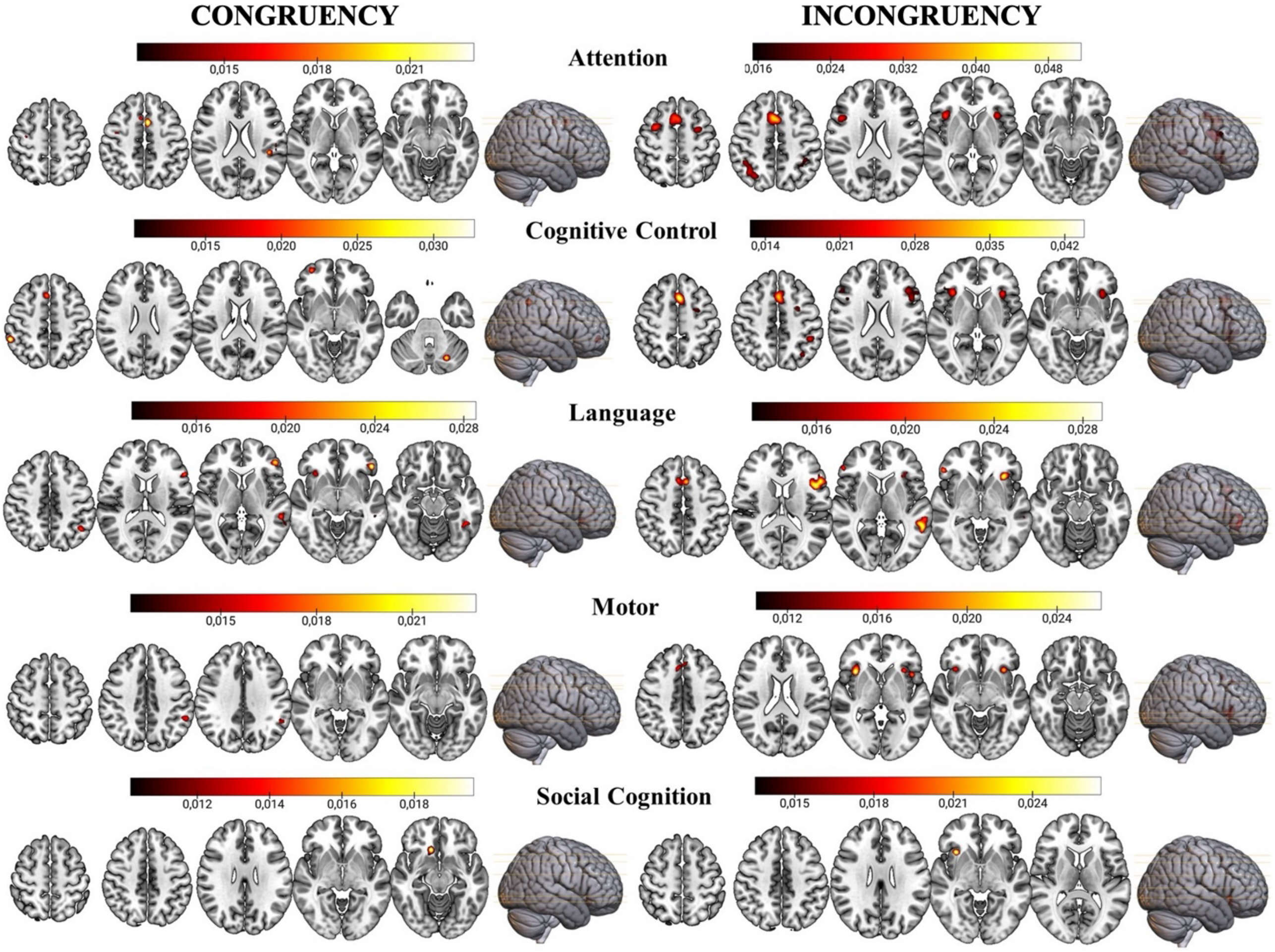


**Fig S1.** Graphical representations of the results for congruency and incongruency processes in the different cognitive domains. Color bar represents ALE values. Images are in radiological convention (R = Right; L = Left).

**Supplementary Information A S3:** Contrast analysis between cross-domain prediction incongruency and cross-domain prediction congruency results

| Cluster | MNI Coordinates | | | Z Value | Region |
|---|---|---|---|---|---|
| **Cross.domain prediction incongruency > Cross-domain prediction congruency** | | | | | |
| 1 | 42.8 | 19.2 | 18 | 3.890 | |
| 1 | 44 | 14 | 20 | 3.719 | *Inferior Frontal Gyrus* |
| 1 | 50 | 22 | 14 | 3.290 | *Middle Frontal Gyrus* |
| 1 | 50 | 12 | 10 | 3.155 | *Insula* |
| 1 | 44 | 8 | 34 | 2.726 | *Precentral Gyrus* |
| 2 | 43.3 | 34 | -6.7 | 3.890 | *Inferior Frontal Gyrus* |
| 2 | 52 | 36 | 2 | 3.719 | *Inferior Frontal Gyrus* |
| 2 | 48 | 36 | 1 | 3.540 | *Inferior Frontal Gyrus* |
| 2 | 50 | 34 | -4 | 3.352 | *Inferior Frontal Gyrus* |
| 2 | 42 | 22 | -8 | 2.967 | *Insula* |
| 2 | 38 | 16 | 4 | 2.669 | *Insula* |
| 2 | 38 | 22 | 4 | 2.635 | *Insula* |
| 2 | 36 | 26 | 4 | 2.627 | *Insula* |
| 3 | -39.7 | 17.7 | 0.7 | 3.890 | *Insula* |
| 4 | -46 | 26 | 30 | 3.890 | *Middle Frontal Gyrus* |
| 5 | -50 | 11 | 19 | 3.121 | *Inferior Frontal Gyrus* |
| 6 | 42 | -60 | 46 | 2.947 | *Inferior Parietal Lobule* |
| 6 | 46 | -58 | 36 | 2.542 | *Angular Gyrus* |

| | | | | | |
|---|---|---|---|---|---|
| 6 | 50 | -62 | 36 | 2.500 | *Middle Temporal Gyrus* |
| 7 | 0 | 12 | 50 | 2.794 | *Superior Frontal Gyrus* |
| 7 | -10 | 8 | 56 | 2.568 | *Medial Frontal Gyrus* |
| 8 | -32 | 2 | 58 | 3.238 | *Middle Frontal Gyrus* |
| 9 | 58 | -28 | -2 | 3.238 | *Superior Temporal Gyrus* |
| 9 | 53 | -28 | -2 | 2.988 | *Superior Temporal Gyrus* |
| **Cross-domain prediction congruency > Cross-domain prediction incongruency** | | | | | |
| 1 | 8.4 | -6 | 47.6 | 3.540 | *Paracentral Lobule* |
| 1 | -3 | -8 | 54 | 2.929 | *Medial Frontal Gyrus* |
| 2 | -37 | -18 | 53 | 3.061 | *Precentral Gyrus* |
| 2 | -40 | -16 | 56 | 3.035 | *Precentral Gyrus* |
| 3 | -54 | -38 | 54 | 3.238 | *Inferior Parietal Lobule* |

**Supplementary Information A S4:** Number of included experiments for each dataset and meta-analysis

Please note that the cross-domain meta-analyses also include experiments from Memory, Music and Pain domains, which did not reach the minimum threshold of 17 experiments to create their own datasets.

| **Phenomena-independent Prediction** | |
|---|---|
| Cross-domain | 252 experiments |
| Attention | 52 experiments |
| Cognitive Control | 31 experiments |
| Language | 53 experiments |
| Motor | 41 experiments |
| Social Cognition | 35 experiments |
| **Prediction Incongruency** | |
| Cross-domain | 175 experiments |
| Attention | 44 experiments |
| Cognitive Control | 21 experiments |
| Language | 39 experiments |
| Motor | 18 experiments |
| Social Cognition | 29 experiments |
| **Prediction Congruency** | |
| Cross-domain | 134 experiments |
| Attention | 17 experiments |
| Cognitive Control | 15 experiments |
| Language | 32 experiments |
| Motor | 28 experiments |
| Social Cognition | 18 experiments |

**Supplementary Information A S5:** Siman-Tov et al., 2019 replication

| Cluster | MNI Coordinates | | | Peak ALE p-value | Region |
|---|---|---|---|---|---|
| **General prediction** | | | | | |
| 1 | 34 | 22 | -2 | 0.041 | *Claustrum* |
| 2 | 50 | 8 | 22 | 0.027 | *Inferior Frontal Gyrus* |
| 2 | 46 | 16 | 14 | 0.022 | *Insula* |
| 2 | 52 | 12 | 12 | 0.021 | *Insula* |
| 3 | -32 | 20 | 2 | 0.036 | *Claustrum* |
| 4 | 2 | 28 | 40 | 0.027 | *Cingulate Gyrus* |
| 5 | -10 | 12 | 2 | 0.032 | *Caudate* |
| 6 | -10 | -80 | -26 | 0.028 | *Pyramis* |
| 7 | -48 | 6 | 22 | 0.026 | *Inferior Frontal Gyrus* |
| 8 | -46 | 22 | 20 | 0.025 | *Middle Frontal Gyrus* |
| 9 | 50 | 16 | -2 | 0.024 | *Insula* |
| 10 | -26 | -8 | 58 | 0.025 | *Precentral Gyrus* |
| 11 | 50 | -32 | 0 | 0.021 | *Superior Temporal Gyrus* |
| 12 | 64 | -26 | 36 | 0.022 | *Inferior Parietal Lobule* |
| 13 | 12 | -12 | -8 | 0.022 | *Subtalamic Nucleus* |

**Supplementary Information A S5:** Siman-Tov et al., 2019 replication

| Cluster | MNI Coordinates | | | Z value | Region |
|---|---|---|---|---|---|
| **Prediction violation > Prediction encoding** | | | | | |
| 1 | 55.6 | -29.2 | -.6 | 3.890 | *Superior Temporal Gyrus* |
| 1 | 55 | -30 | 3 | 0 | *Superior Temporal Gyrus* |
| 1 | 52 | -36 | 0 | 3.035 | *Superior Temporal Gyrus* |
| **Prediction encoding > Prediction violation** | | | | | |
| No significant results | | | | | |

**Supplementary Information A S6:** Seed-based d Mapping Meta-Analyses

## METHODS

Meta-analyses were carried out also using Seed-based d Mapping with Permutation of Subject Images (SDM-PSI, v6.22, https://www.sdmproject.com/ (Albajes-Eizagirre et al., 2019a; Albajes-Eizagirre et al., 2019b)). Please note that the cross-domain prediction phenomena-independent, prediction incongruency, and prediction congruency meta-analyses were not performed due to constant machine crashing caused by the high computational demand. For experiments using any indicator of significance other than t-values, the online utilities provided by the developers of SDM-PSI (https://www.sdmproject.com/utilities/) were used to convert them into t-values.

The algorithm used by SDM-PSI estimates the lower and upper bounds of possible effect size images from the t-values associated with each focus from the included studies. Afterwards, SDM uses MetaNSUE (a meta-analytic method that allows an unbiased inclusion of studies with Non-statistically Significant Unreported Effects by using maximum likelihood estimation and multiple imputation techniques) to estimate the most likely effect size and standard error and executes multiple imputations to add noise to the estimations within the previously established bounds. SDM-PSI then performs a meta-analysis on each imputed dataset and combines the imputed meta-analyzed datasets with Rubin's rules. Finally subject images are recreated to run standard permutations tests, in which the distribution of maxima is used to correct for multiple comparisons by means of Family-Wise Error (FWE) Rate.

All meta-analyses were conducted as follows:

1. Data were preprocessed using the native preprocessing function of SDM-PSI.
2. The actual meta-analysis was conducted with the method described above.
3. The results were corrected for multiple comparisons using FWE and 1,000 permutations.
4. The corrected results were thresholded to identify significant clusters, with an extent threshold of 100 voxels.
5. A mask was created for every significantly active cluster using the MNI coordinates provided at thresholding.
6. The mask was used to extract the Hedges' g statistic linked to that specific cluster.

## RESULTS

| Cluster | MNI Coordinates | SDM-Z values | Region |
|---|---|---|---|
| **Attention** | | | |
| 1 | 2 8 56 | 10.115 | Supplementary motor area |
| **Cognitive Control** | | | |
| 1 | 2 18 48 | 10.894 | Supplementary motor area |
| 2 | -28 16 -6 | 7.294 | Insula |
| 3 | 42 20 2 | 5.745 | Insula |
| 4 | -52 -42 42 | 9.166 | Inferior parietal gyri |
| 5 | 48 -46 46 | 7.770 | Inferior parietal gyri |
| 6 | -30 -68 -26 | 6.407 | Cerebellum, lobule VI |
| 7 | 0 -34 32 | 6.471 | Posterior cingulate |
| **Language** | | | |
| 1 | -50 22 6 | 8.096 | Inferior frontal gyrus, triangular part |
| 2 | 56 -18 12 | 7.420 | Rolandic operculum |
| 3 | 0 8 40 | 6.266 | Median cingulate |
| **Motor** | | | |
| 1 | 28 18 0 | 2.279 | Lenticular nucleus, putamen |
| 2 | 2 16 42 | 6.541 | Medial superior frontal gyrus |
| 3 | -50 -48 38 | 5.279 | Inferior frontal gyrus |
| 4 | -34 10 0 | 4.797 | Insula |
| 5 | -6 0 4 | 4.950 | *Undefined* (local peaks in the anterior thalamic projections and caudate nucleus) |
| **Social Cognition** | | | |
| 1 | 38 20 -8 | 6.715 | Insula |
| 2 | 2 48 0 | 5.199 | Medial superior frontal gyrus |
| 3 | 36 -52 -24 | 4.713 | Cerebellum, lobule VI |
| 4 | 0 -46 36 | 3.771 | Medial cingulate |
| 5 | -54 -26 36 | 3.724 | Inferior parietal gyrus |

**Table S1.** Results of the domain-specific prediction phenomena-independent meta-analyses run on SDM.

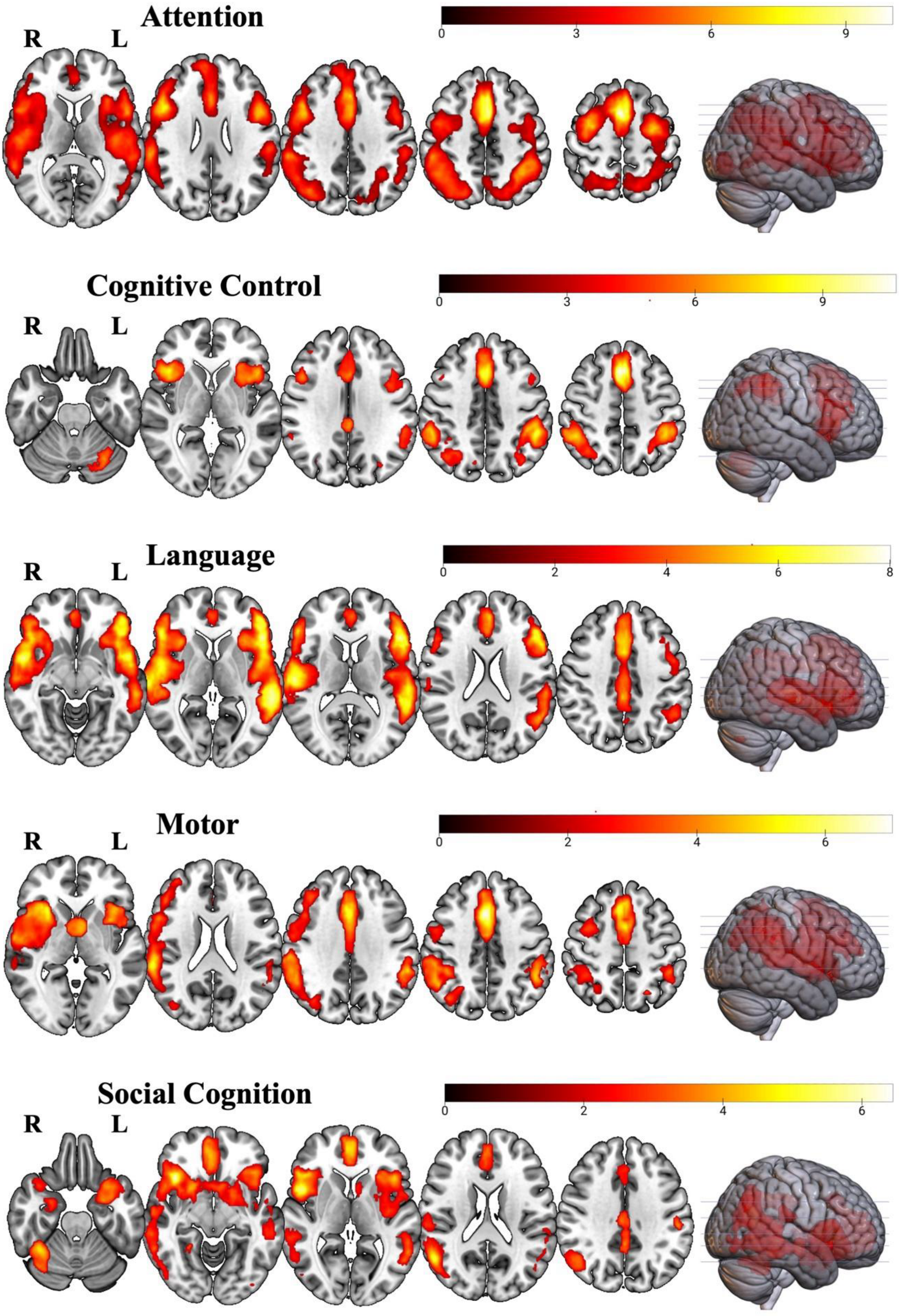


**Figure S2.** Main clusters of activation in all domain-specific prediction phenomena-independent meta-analyses run on SDM. Color bars refer to SDM-Z values. Images are in radiological convention (R = Right; L = Left).

| **Congruency** | | | | **Incongruency** | | | |
|---|---|---|---|---|---|---|---|
| **Cluster** | **MNI Coordinates** | **SDM-Z values** | **Region** | **Cluster** | **MNI Coordinates** | **SDM-Z values** | **Region** |
| **Attention** | | | | | | | |
| 1 | 2 4 52 | 6.494 | Supplementary motor area | 1 | 4 8 56 | 8.644 | Supplementary motor area |
| 2 | 40 -80 -10 | 3.042 | Inferior occipital gyrus | 2 | -48 8 26 | 5.384 | Inferior frontal gyrus, opercular part |
| | | | | 3 | -56 -38 12 | 4.342 | Superior longitudinal fasciculus III |
| | | | | 4 | -50 -46 44 | 5.487 | Inferior parietal gyrus |
| **Cognitive Control** | | | | | | | |
| 1 | -30 -70 -30 | 6.944 | Cerebellum, crus I | 1 | -4 20-50 | 7.922 | Supplementary motor area |
| 2 | -46 -38 46 | 6.091 | Inferior parietal gyri | 2 | -46 18 4 | 6.189 | Inferior frontal gyrus, triangular part |
| 3 | 6 20 54 | 6.395 | Supplementary motor area | 3 | 40 20 -4 | 3.548 | Insula |
| 4 | 54 -48 46 | 6.118 | Inferior parietal gyri | 4 | -48 -36 44 | 5.693 | Inferior parietal gyrus |
| 5 | 0 -32 32 | 5.933 | Posterior cingulate | 5 | 46 12 32 | 5.360 | Inferior frontal gyrus, opercular gyrus |
| 6 | 50 24 2 | 4.990 | Inferior frontal gyrus, triangular part | | | | |
| **Language** | | | | | | | |
| 1 | -50 32 2 | 7.636 | Inferior frontal gyrus, triangular part | 1 | -48 28 20 | 7.106 | Inferior frontal gyrus, triangular part |
| 2 | 58 -18 10 | 7.141 | Superior temporal gyrus | 2 | 52 30 10 | 2.470 | Inferior frontal gyrus, triangular part |
| 3 | 2 -36 38 | 6.516 | Median cingulate | 3 | -2 42 24 | 1.036 | Medial superior frontal gyrus |

| | | | | | | | |
|---|---|---|---|---|---|---|---|
| 4 | 30 -74 -40 | 4.990 | Cerebellum, crus II | | | | |
| **Motor** | | | | | | | |
| 1 | 4 18 32 | 4.810 | Median cingulate gyrus | 1 | 62 -10 10 | 5.185 | Rolandic operculum |
| 2 | -50 48 38 | 5.497 | Inferior parietal gyrus | 2 | 4 18 50 | 5.845 | Supplementary motor area |
| 3 | 58 -46 28 | 4.818 | Supramarginal gyrus | | | | |
| 4 | 26 -2 -8 | 5.249 | Striatum | | | | |
| 5 | -8 2 2 | 5.995 | *Undefined* (local peaks in the anterior thalamic projections and corpus callosum) | | | | |
| 6 | 34 34 34 | 0.197 | Middle frontal gyrus | | | | |
| **Social Cognition** | | | | | | | |
| *No significant results* | | | | 1 | 0 28 38 | 5.869 | Medial superior frontal gyrus |
| | | | | 2 | 50 8 2 | 5.944 | Rolandic operculum |
| | | | | 3 | 50 -64 26 | 5.524 | Angular gyrus |
| | | | | 4 | 2 50 0 | 4.711 | Medial superior frontal gyrus |
| | | | | 5 | 0 -48 40 | 4.632 | Precuneus |
| | | | | 6 | 38 -50 -24 | 5.307 | Fusiform gyrus |

**Table S2.** Results of the domain-specific prediction congruency and incongruency meta-analyses run on SDM.

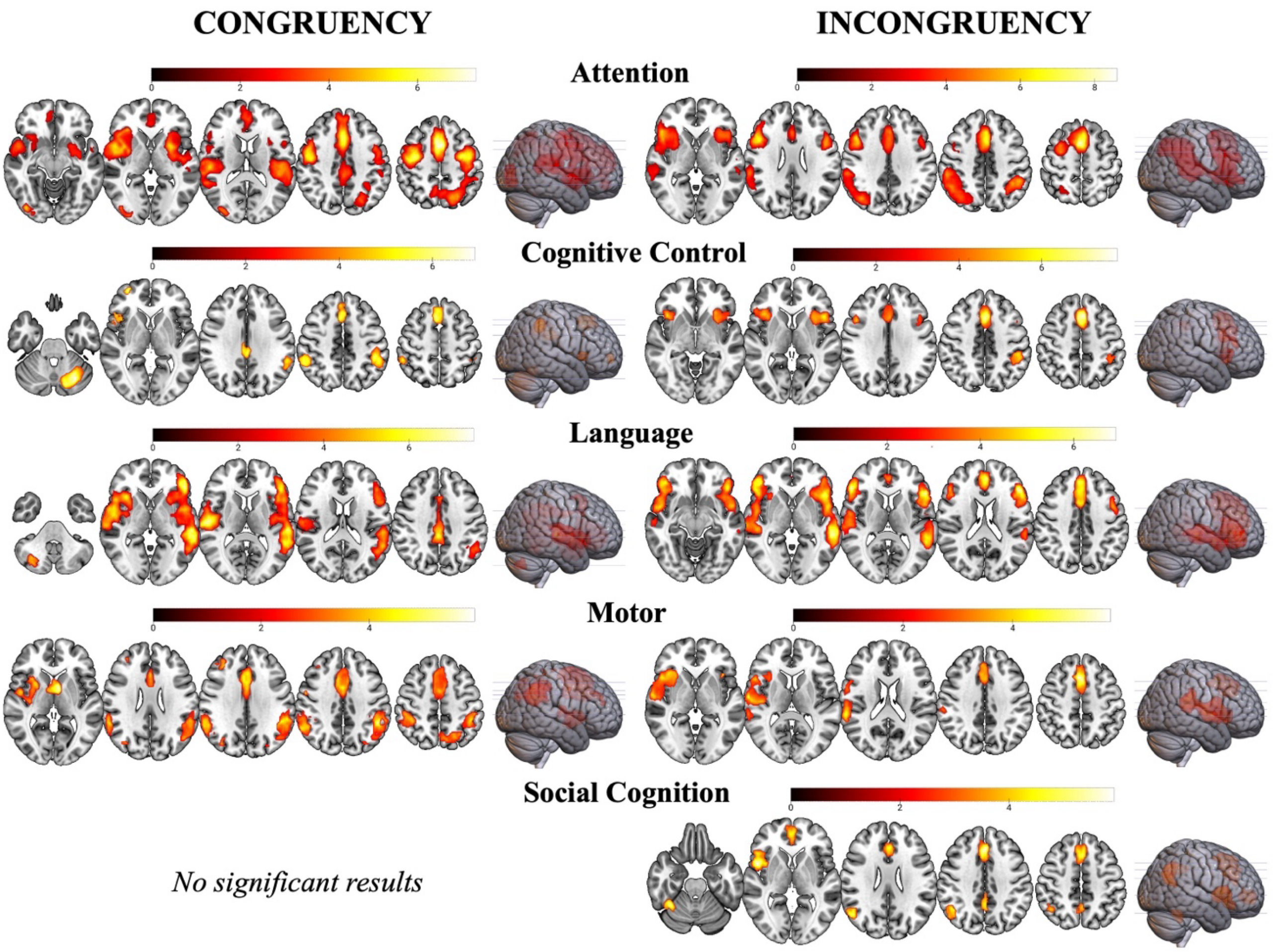


**Figure S3.** Main clusters of activation in all prediction congruency and incongruency meta-analyses run on SDM. Color bars refer to SDM-Z values. Images are in radiological convention (R = Right; L = Left).

| Phenomena-independent | | | Congruency | | Incongruency | |
|---|---|---|---|---|---|---|
| **Cluster** | **$I^2$** | **Excess Sign. (p)** | **$I^2$** | **Excess Sign. (p)** | **$I^2$** | **Excess Sign. (p)** |
| **Attention** | | | | | | |
| 1 | 21.705 | .972 | 2.701 | .772 | 23.776 | .820 |
| 2 | | | 26.756 | .447 | 27.644 | 1.000 |
| 3 | | | | | 18.717 | .260 |
| 4 | | | | | 6.244 | .989 |
| **Cognitive control** | | | | | | |
| 1 | 4.267 | .854 | 4.100 | .993 | 4.648 | .730 |
| 2 | 18.336 | .854 | 7.513 | .628 | 4.845 | .997 |
| 3 | 43.558 | 1.000 | 5.513 | .886 | 70.639 | .924 |
| 4 | 6.014 | .960 | 12.615 | .973 | 12.432 | .637 |
| 5 | 2.521 | .955 | 0.967 | .611 | 2.573 | 1.000 |
| 6 | 5.678 | .974 | 1.100 | <.001* | | |
| 7 | 0.649 | .988 | | | | |
| **Language** | | | | | | |
| 1 | 5.171 | .561 | 5.864 | .966 | 4.393 | 1.000 |
| 2 | 32.622 | <.001* | 0.893 | .434 | 59.519 | 1.000 |
| 3 | 34.721 | 1.000 | 1.621 | .995 | 3.471 | 1.000 |
| 4 | | | 48.255 | .750 | | |
| **Motor** | | | | | | |
| 1 | 63.473 | .771 | 39.178 | .995 | 18.168 | .410 |
| 2 | 40.288 | .994 | 46.349 | .726 | 6.806 | .655 |
| 3 | 39.670 | .951 | 5.275 | .933 | | |
| 4 | 21.507 | .370 | 0.482 | .233 | | |
| 5 | 62.659 | .702 | 5.430 | .643 | | |
| 6 | | | 0.631 | .599 | | |
| **Social cognition** | | | | | | |
| 1 | 5.519 | .784 | | | 4.694 | .943 |
| 2 | 6.660 | .950 | | | 2.351 | .876 |
| 3 | 2.007 | .776 | | | 8.858 | .971 |
| 4 | 2.004 | .382 | | | 22.637 | .362 |
| 5 | 25.065 | .688 | | | 0.185 | .695 |
| 6 | | | | | 0.518 | .955 |

**Table S3.** $I^2$ values of between-study heterogeneity; Excess Significance Test p value for publication bias.

## References

Albajes-Eizagirre, A., Solanes, A., Fullana, M. A., Ioannidis, J. P., Fusar-Poli, P., Torrent, C., ... & Radua, J. (2019a). Meta-analysis of voxel-based neuroimaging studies using seed-based d mapping with permutation of subject images (SDM-PSI). *JoVE (Journal of Visualized Experiments)*, (153), e59841.

Albajes-Eizagirre, A., Solanes, A., Vieta, E., & Radua, J. (2019b). Voxel-based meta-analysis via permutation of subject images (PSI): Theory and implementation for SDM. *Neuroimage*, *186*, 174-184.

**Supplementary Information A S7:** References of studies included in the meta-analyses


Aben, B., Calderon, C. B., Van der Cruyssen, L., Picksak, D., Van den Bussche, E., & Verguts, T. (2019). Context-dependent modulation of cognitive control involves different temporal profiles of fronto-parietal activity. NeuroImage, 189, 755-762.

Alderson-Day, B., Lima, C. F., Evans, S., Krishnan, S., Shanmugalingam, P., Fernyhough, C., & Scott, S. K. (2017). Distinct processing of ambiguous speech in people with non-clinical auditory verbal hallucinations. Brain, 140(9), 2475-2489.

Alho, K., Salmi, J., Koistinen, S., Salonen, O., & Rinne, T. (2015). Top-down controlled and bottom-up triggered orienting of auditory attention to pitch activate overlapping brain networks. Brain research, 1626, 136-145.

Alvarez, T. L., Alkan, Y., Gohel, S., Ward, B. D., & Biswal, B. B. (2010). Functional anatomy of predictive vergence and saccade eye movements in humans: a functional MRI investigation. Vision research, 50(21), 2163-2175.

Andics, A., Gál, V., Vicsi, K., Rudas, G., & Vidnyánszky, Z. (2013). FMRI repetition suppression for voices is modulated by stimulus expectations. NeuroImage, 69, 277–283.

Arrington, C. M., Carr, T. H., Mayer, A. R., & Rao, S. M. (2000). Neural mechanisms of visual attention: object-based selection of a region in space. Journal of cognitive neuroscience, 12(Supplement 2), 106-117.

Atmaca, S., Stadler, W., Keitel, A., Ott, D. V., Lepsien, J., & Prinz, W. (2013). Prediction processes during multiple object tracking (MOT): involvement of dorsal and ventral premotor cortices. Brain and behavior, 3(6), 683-700.

Aue, T., Guex, R., Chauvigné, L. A., Okon-Singer, H., & Vuilleumier, P. (2019). Expectancies influence attention to neutral but not necessarily to threatening stimuli: An fMRI study. Emotion, 19(7), 1244.

Bardi, L., Desmet, C., Nijhof, A., Wiersema, J. R., & Brass, M. (2017). Brain activation for spontaneous and explicit false belief tasks overlaps: new fMRI evidence on belief processing and violation of expectation. Social cognitive and affective neuroscience, 12(3), 391-400.

Beldzik, E., Domagalik, A., Oginska, H., Marek, T., & Fafrowicz, M. (2015). Brain activations related to saccadic response conflict are not sensitive to time on task. Frontiers in Human Neuroscience, 9, 664.

Bengtsson, S. L., Ullen, F., Ehrsson, H. H., Hashimoto, T., Kito, T., Naito, E., ... & Sadato, N. (2009). Listening to rhythms activates motor and premotor cortices. Cortex, 45(1), 62-71.

Benn, Y., Webb, T. L., Chang, B. P., Sun, Y. H., Wilkinson, I. D., & Farrow, T. F. (2014). The neural basis of monitoring goal progress. Frontiers in Human Neuroscience, 8, 688.

Bianco, R., Novembre, G., Keller, P. E., Kim, S. G., Scharf, F., Friederici, A. D., ... & Sammler, D. (2016). Neural networks for harmonic structure in music perception and action. Neuroimage, 142, 454-464.

Blank, H., & von Kriegstein, K. (2013). Mechanisms of enhancing visual–speech recognition by prior auditory information. Neuroimage, 65, 109-118.

Blank, H., Spangenberg, M., & Davis, M. H. (2018). Neural prediction errors distinguish perception and misperception of speech. Journal of Neuroscience, 38(27), 6076-6089.

Blank, H., & Davis, M. H. (2016). Prediction errors but not sharpened signals simulate multivoxel fMRI patterns during speech perception. PLoS biology, 14(11), e1002577.

Böckler, A., Eskenazi, T., Sebanz, N., & Rueschemeyer, S. A. (2016). (How) observed eye-contact modulates gaze following. An fMRI study. Cognitive neuroscience, 7(1-4), 55-66.

Bohrn, I. C., Altmann, U., Lubrich, O., Menninghaus, W., & Jacobs, A. M. (2012). Old proverbs in new skins–an fMRI study on defamiliarization. Frontiers in psychology, 3, 204.

Bonhage, C. E., Mueller, J. L., Friederici, A. D., & Fiebach, C. J. (2015). Combined eye tracking and fMRI reveals neural basis of linguistic predictions during sentence comprehension. Cortex, 68, 33-47.

Boorman, E. D., O'Doherty, J. P., Adolphs, R., & Rangel, A. (2013). The behavioral and neural mechanisms underlying the tracking of expertise. Neuron, 80(6), 1558-1571.

Cacciaglia, R., Costa-Faidella, J., Zarnowiec, K., Grimm, S., & Escera, C. (2019). Auditory predictions shape the neural responses to stimulus repetition and sensory change. Neuroimage, 186, 200-210.

Cacioppo, S., Fontang, F., Patel, N., Decety, J., Monteleone, G., & Cacioppo, J. T. (2014). Intention understanding over T: a neuroimaging study on shared representations and tennis return predictions. Frontiers in human neuroscience, 8, 781.

Cacioppo, S., Juan, E., & Monteleone, G. (2017). Predicting intentions of a familiar significant other beyond the mirror neuron system. Frontiers in behavioral neuroscience, 11, 155.

Campbell, M. E., Nguyen, V. T., Cunnington, R., & Breakspear, M. (2021). Insula cortex gates the interplay of action observation and preparation for controlled imitation. Neuropsychologia, 161, 108021.

Carp, J., Kim, K., Taylor, S. F., Fitzgerald, K. D., & Weissman, D. H. (2010). Conditional differences in mean reaction time explain effects of response congruency, but not accuracy, on posterior medial frontal cortex activity. Frontiers in Human Neuroscience, 4, 231.

Carter, C. S., Mintun, M., & Cohen, J. D. (1995). Interference and facilitation effects during selective attention: an H215O PET study of Stroop task performance. Neuroimage, 2(4), 264-272.

Carvalho, F. M., Chaim, K. T., Sanchez, T. A., & De Araujo, D. B. (2016). Time-perception network and default mode network are associated with temporal prediction in a periodic motion task. Frontiers in human neuroscience, 268.

Cassidy, B. S., & Gutchess, A. H. (2015). Neural responses to appearance-behavior congruity. Social cognition, 33(3), 211-226.

Cazzato, V., Macaluso, E., Crostella, F., & Aglioti, S. M. (2012). Mapping reflexive shifts of attention in eye-centered and hand-centered coordinate systems. Human brain mapping, 33(1), 165-178.

Chaminade, T., Meary, D., Orliaguet, J. P., & Decety, J. (2001). Is perceptual anticipation a motor simulation? A PET study. NeuroReport, 12(17), 3669-3674.

Chen, S. A., & Desmond, J. E. (2005). Cerebrocerebellar networks during articulatory rehearsal and verbal working memory tasks. Neuroimage, 24(2), 332-338.

Chiu, Y. C., Jiang, J., & Egner, T. (2017). The caudate nucleus mediates learning of stimulus–control state associations. Journal of Neuroscience, 37(4), 1028-1038.

Chou, T. L., Lee, S. H., Hung, S. M., & Chen, H. C. (2012). The role of inferior frontal gyrus in processing Chinese classifiers. Neuropsychologia, 50(7), 1408-1415.

Christensen, T. A., Lockwood, J. L., Almryde, K. R., & Plante, E. (2011). Neural substrates of attentive listening assessed with a novel auditory Stroop task. Frontiers in human neuroscience, 4, 236.

Christopoulos, G. I., & King-Casas, B. (2015). With you or against you: Social orientation dependent learning signals guide actions made for others. NeuroImage, 104, 326-335.

Clos, M., Langner, R., Meyer, M., Oechslin, M. S., Zilles, K., & Eickhoff, S. B. (2014). Effects of prior information on decoding degraded speech: an fMRI study. Human brain mapping, 35(1), 61-74.

Cloutier, J., Gabrieli, J. D., O'young, D., & Ambady, N. (2011). An fMRI study of violations of social expectations: when people are not who we expect them to be. NeuroImage, 57(2), 583-588.

Cohen, A. O., Dellarco, D. V., Breiner, K., Helion, C., Heller, A. S., Rahdar, A., ... & Casey, B. J. (2016). The impact of emotional states on cognitive control circuitry and function. Journal of cognitive neuroscience, 28(3), 446-459.

Collins, A. G., Ciullo, B., Frank, M. J., & Badre, D. (2017). Working memory load strengthens reward prediction errors. Journal of Neuroscience, 37(16), 4332-4342.

Cooke, A., Grossman, M., DeVita, C., Gonzalez-Atavales, J., Moore, P., Chen, W., ... & Detre, J. (2006). Large-scale neural network for sentence processing. Brain and language, 96(1), 14-36.

Cotti, J., Rohenkohl, G., Stokes, M., Nobre, A. C., & Coull, J. T. (2011). Functionally dissociating temporal and motor components of response preparation in left intraparietal sulcus. Neuroimage, 54(2), 1221-1230.

Coull, J. T., Cotti, J., & Vidal, F. (2016). Differential roles for parietal and frontal cortices in fixed versus evolving temporal expectations: Dissociating prior from posterior temporal probabilities with fMRI. Neuroimage, 141, 40-51.

Crafa, D., Hawco, C., & Brodeur, M. B. (2017). Heightened responses of the parahippocampal and retrosplenial cortices during contextualized recognition of congruent objects. Frontiers in behavioral neuroscience, 11, 232.

Criaud, M., Longcamp, M., Anton, J. L., Nazarian, B., Roth, M., Sescousse, G., ... & Boulinguez, P. (2017). Testing the physiological plausibility of conflicting psychological models of response inhibition: a forward inference fMRI study. Behavioural Brain Research, 333, 192-202.

Cross, E. S., Stadler, W., Parkinson, J., Schütz-Bosbach, S., & Prinz, W. (2013). The influence of visual training on predicting complex action sequences. Human brain mapping, 34(2), 467-486.

Danek, A. H., Öllinger, M., Fraps, T., Grothe, B., & Flanagin, V. L. (2015). An fMRI investigation of expectation violation in magic tricks. Frontiers in psychology, 6, 84.

Danielsen, A., Otnæss, M. K., Jensen, J., Williams, S. C. R., & Østberg, B. C. (2014). Investigating repetition and change in musical rhythm by functional MRI. Neuroscience, 275, 469-476.

Davis, B., & Hasson, U. (2018). Predictability of what or where reduces brain activity, but a bottleneck occurs when both are predictable. NeuroImage, 167, 224-236.

D'Cruz, A. M., Ragozzino, M. E., Mosconi, M. W., Pavuluri, M. N., & Sweeney, J. A. (2011). Human reversal learning under conditions of certain versus uncertain outcomes. Neuroimage, 56(1), 315-322.

den Ouden, H. E., Friston, K. J., Daw, N. D., McIntosh, A. R., & Stephan, K. E. (2009). A dual role for prediction error in associative learning. Cerebral cortex, 19(5), 1175-1185.

den Ouden, H. E., Daunizeau, J., Roiser, J., Friston, K. J., & Stephan, K. E. (2010). Striatal prediction error modulates cortical coupling. Journal of Neuroscience, 30(9), 3210-3219.

Diaconescu, A. O., Mathys, C., Weber, L. A., Kasper, L., Mauer, J., & Stephan, K. E. (2017). Hierarchical prediction errors in midbrain and septum during social learning. Social cognitive and affective neuroscience, 12(4), 618-634.

Diekhof, E. K., Kipshagen, H. E., Falkai, P., Dechent, P., Baudewig, J., & Gruber, O. (2011). The power of imagination—How anticipatory mental imagery alters perceptual processing of fearful facial expressions. NeuroImage, 54(2), 1703-1714.

Dietrich, S., Hertrich, I., Seibold, V. C., & Rolke, B. (2019). Discourse management during speech perception: A functional magnetic resonance imaging (fMRI) study. NeuroImage, 202, 116047.

Domahs, U., Klein, E., Huber, W., & Domahs, F. (2013). Good, bad and ugly word stress–fMRI evidence for foot structure driven processing of prosodic violations. Brain and language, 125(3), 272-282.

Dombert, P. L., Kuhns, A., Mengotti, P., Fink, G. R., & Vossel, S. (2016). Functional mechanisms of probabilistic inference in feature-and space-based attentional systems. NeuroImage, 142, 553-564.

Dungan, J. A., Stepanovic, M., & Young, L. (2016). Theory of mind for processing unexpected events across contexts. Social Cognitive and Affective Neuroscience, 11(8), 1183-1192.

Dunne, L., & Opitz, B. (2020). Attention control processes that prioritise task execution may come at the expense of incidental memory encoding. Brain and cognition, 144, 105602.

Dzafic, I., Martin, A. K., Hocking, J., Mowry, B., & Burianová, H. (2016). Dynamic emotion perception and prior expectancy. Neuropsychologia, 86, 131-140.

Eickhoff, S. B., Pomjanski, W., Jakobs, O., Zilles, K., & Langner, R. (2011). Neural correlates of developing and adapting behavioral biases in speeded choice reactions—an fMRI study on predictive motor coding. Cerebral cortex, 21(5), 1178-1191.

Fajkus, J., Mikl, M., Shaw, D. J., & Brázdil, M. (2015). An fMRI investigation into the effect of preceding stimuli during visual oddball tasks. Journal of Neuroscience Methods, 251, 56-61.

Fareri, D. S., Chang, L. J., & Delgado, M. R. (2012). Effects of direct social experience on trust decisions and neural reward circuitry. Frontiers in neuroscience, 6, 148.

Farmer, H., Hertz, U., & Hamilton, A. F. D. C. (2019). The neural basis of shared preference learning. Social Cognitive and Affective Neuroscience, 14(10), 1061-1072.

Fenker, D. B., Schoenfeld, M. A., Waldmann, M. R., Schuetze, H., Heinze, H. J., & Duezel, E. (2010). "Virus and epidemic": causal knowledge activates prediction error circuitry. Journal of cognitive neuroscience, 22(10), 2151-2163.

Flegal, K. E., Marín-Gutiérrez, A., Ragland, J. D., & Ranganath, C. (2014). Brain mechanisms of successful recognition through retrieval of semantic context. Journal of Cognitive Neuroscience, 26(8), 1694-1704.

Foudil, S. A., Kwok, S. C., & Macaluso, E. (2020). Context-dependent coding of temporal distance between cinematic events in the human precuneus. Journal of Neuroscience, 40(10), 2129-2138.

Friederici, A. D., Kotz, S. A., Scott, S. K., & Obleser, J. (2010). Disentangling syntax and intelligibility in auditory language comprehension. Human brain mapping, 31(3), 448-457.

Friedman, D., Goldman, R., Stern, Y., & Brown, T. R. (2009). The brain's orienting response: An event-related functional magnetic resonance imaging investigation. Human brain mapping, 30(4), 1144-1154.

Frühholz, S., Fehr, T., & Herrmann, M. (2009). Interference control during recognition of facial affect enhances the processing of expression specific properties—An event-related fMRI study. Brain research, 1269, 143-157.

Gagnepain, P., Henson, R., Chételat, G., Desgranges, B., Lebreton, K., & Eustache, F. (2011). Is neocortical–hippocampal connectivity a better predictor of subsequent recollection than local increases in hippocampal activity? New insights on the role of priming. Journal of Cognitive Neuroscience, 23(2), 391-403.

Gardner, T., Goulden, N., & Cross, E. S. (2015). Dynamic modulation of the action observation network by movement familiarity. Journal of Neuroscience, 35(4), 1561-1572.

Gayet, S., Guggenmos, M., Christophel, T. B., Haynes, J. D., Paffen, C. L., Van der Stigchel, S., & Sterzer, P. (2017). Visual working memory enhances the neural response to matching visual input. Journal of Neuroscience, 37(28), 6638-6647.

Gertz, H., Hilger, M., Hegele, M., & Fiehler, K. (2016). Violating instructed human agency: An fMRI study on ocular tracking of biological and nonbiological motion stimuli. Neuroimage, 138, 109-122.

Geuter, S., Boll, S., Eippert, F., & Büchel, C. (2017). Functional dissociation of stimulus intensity encoding and predictive coding of pain in the insula. Elife, 6, e24770.

Gordon, I., Eilbott, J. A., Feldman, R., Pelphrey, K. A., & Vander Wyk, B. C. (2013). Social, reward, and attention brain networks are involved when online bids for joint attention are met with congruent versus incongruent responses. Social neuroscience, 8(6), 544-554.

Gottfried, J. A., & Dolan, R. J. (2003). The nose smells what the eye sees: crossmodal visual facilitation of human olfactory perception. Neuron, 39(2), 375-386.

Grahn, J. A., & Rowe, J. B. (2009). Feeling the beat: premotor and striatal interactions in musicians and nonmusicians during beat perception. Journal of Neuroscience, 29(23), 7540-7548.

Grahn, J. A., & Rowe, J. B. (2013). Finding and feeling the musical beat: striatal dissociations between detection and prediction of regularity. Cerebral cortex, 23(4), 913-921.

Grewe, T., Bornkessel-Schlesewsky, I., Zysset, S., Wiese, R., Von Cramon, D. Y., & Schlesewsky, M. (2007). The role of the posterior superior temporal sulcus in the processing of unmarked transitivity. NeuroImage, 35(1), 343-352.

Grotheer, M., & Kovács, G. (2015). The relationship between stimulus repetitions and fulfilled expectations. Neuropsychologia, 67, 175-182.

Grotheer, M., Hermann, P., Vidnyánszky, Z., & Kovács, G. (2014). Repetition probability effects for inverted faces. NeuroImage, 102, 416-423.

Gruber, O., Melcher, T., Diekhof, E. K., Karch, S., Falkai, P., & Goschke, T. (2009). Brain mechanisms associated with background monitoring of the environment for potentially significant sensory events. Brain and cognition, 69(3), 559-564.

Gu, Y., Hu, X., Pan, W., Yang, C., Wang, L., Li, Y., & Chen, A. (2016). Neural activities underlying the feedback express salience prediction errors for appetitive and aversive stimuli. Scientific reports, 6(1), 34032.

Hagura, N., Oouchida, Y., Aramaki, Y., Okada, T., Matsumura, M., Sadato, N., & Naito, E. (2009). Visuokinesthetic perception of hand movement is mediated by cerebro–cerebellar interaction between the left cerebellum and right parietal cortex. Cerebral Cortex, 19(1), 176-186.

Hakonen, M., May, P. J., Jääskeläinen, I., Jokinen, E., Sams, M., & Tiitinen, H. (2017). Predictive processing increases intelligibility of acoustically distorted speech.

Ham, T., Leff, A., de Boissezon, X., Joffe, A., & Sharp, D. J. (2013). Cognitive control and the salience network: an investigation of error processing and effective connectivity. Journal of Neuroscience, 33(16), 7091-7098.

Harlé, K. M., Bomyea, J., Spadoni, A. D., Simmons, A. N., & Taylor, C. T. (2020). Proactive engagement of cognitive control modulates implicit approach-avoidance bias. Cognitive, Affective, & Behavioral Neuroscience, 20, 998-1010.

Harris, L. T., & Fiske, S. T. (2010). Neural regions that underlie reinforcement learning are also active for social expectancy violations. Social neuroscience, 5(1), 76-91.

Haupt, S., Axmacher, N., Cohen, M. X., Elger, C. E., & Fell, J. (2009). Activation of the caudal anterior cingulate cortex due to task-related interference in an auditory Stroop paradigm. Human brain mapping, 30(9), 3043-3056.

Heil, L., Colizoli, O., Hartstra, E., Kwisthout, J., van Pelt, S., van Rooij, I., & Bekkering, H. (2019). Processing of prediction errors in mentalizing areas. Journal of Cognitive Neuroscience, 31(6), 900-912.

Heim, S., Friederici, A. D., Schiller, N. O., Rüschemeyer, S. A., & Amunts, K. (2009). The determiner congruency effect in language production investigated with functional MRI. Human brain mapping, 30(3), 928-940.

Heim, S., van Ermingen, M., Huber, W., & Amunts, K. (2010). Left cytoarchitectonic BA 44 processes syntactic gender violations in determiner phrases. Human brain mapping, 31(10), 1532-1541.

Henco, L., Brandi, M. L., Lahnakoski, J. M., Diaconescu, A. O., Mathys, C., & Schilbach, L. (2020). Bayesian modelling captures inter-individual differences in social belief computations in the putamen and insula. cortex, 131, 221-236.

Henderson, L. A., Di Pietro, F., Youssef, A. M., Lee, S., Tam, S., Akhter, R., ... & Macey, P. M. (2020). Effect of expectation on pain processing: A psychophysics and functional MRI analysis. Frontiers in neuroscience, 14, 6.

Henderson, J. M., Choi, W., Lowder, M. W., & Ferreira, F. (2016). Language structure in the brain: A fixation-related fMRI study of syntactic surprisal in reading. Neuroimage, 132, 293-300.

Hillebrandt, H., Dumontheil, I., Blakemore, S. J., & Roiser, J. P. (2013). Dynamic causal modelling of effective connectivity during perspective taking in a communicative task. Neuroimage, 76, 116-124.

Hoenig, K., & Scheef, L. (2005). Mediotemporal contributions to semantic processing: fMRI evidence from ambiguity processing during semantic context verification. Hippocampus, 15(5), 597-609.

Hoenig, K., & Scheef, L. (2009). Neural correlates of semantic ambiguity processing during context verification. Neuroimage, 45(3), 1009-1019.

Holloway, I. D., van Atteveldt, N., Blomert, L., & Ansari, D. (2015). Orthographic dependency in the neural correlates of reading: evidence from audiovisual integration in English readers. Cerebral Cortex, 25(6), 1544-1553.

Hu, S., Ide, J. S., Zhang, S., & Chiang-shan, R. L. (2015). Anticipating conflict: neural correlates of a Bayesian belief and its motor consequence. Neuroimage, 119, 286-295.

Hu, J., Hu, S., Maisano, J. R., Chao, H. H., Zhang, S., & Li, C. S. R. (2016). Novelty seeking, harm avoidance, and cerebral responses to conflict anticipation: An exploratory study. Frontiers in human neuroscience, 10, 546.

Hu, S., Ide, J. S., Zhang, S., & Chiang-shan, R. L. (2016). The right superior frontal gyrus and individual variation in proactive control of impulsive response. Journal of Neuroscience, 36(50), 12688-12696.

Huang, S., Belliveau, J. W., Tengshe, C., & Ahveninen, J. (2012). Brain networks of novelty-driven involuntary and cued voluntary auditory attention shifting.

Huang, J., Zhu, Z., Zhang, J. X., Wu, M., Chen, H. C., & Wang, S. (2012). The role of left inferior frontal gyrus in explicit and implicit semantic processing. Brain research, 1440, 56-64.

Hubers, F., Snijders, T. M., & De Hoop, H. (2016). How the brain processes violations of the grammatical norm: An fMRI study. Brain and Language, 163, 22-31.

Ikeda, Y., Yahata, N., Takahashi, H., Koeda, M., Asai, K., Okubo, Y., & Suzuki, H. (2010). Cerebral activation associated with speech sound discrimination during the diotic listening task: an fMRI study. Neuroscience Research, 67(1), 65-71.

Jakobs, O., Wang, L. E., Dafotakis, M., Grefkes, C., Zilles, K., & Eickhoff, S. B. (2009). Effects of timing and movement uncertainty implicate the temporo-parietal junction in the prediction of forthcoming motor actions. Neuroimage, 47(2), 667-677.

Johnson, M. A., Turk-Browne, N. B., & Goldberg, A. E. (2016). Neural systems involved in processing novel linguistic constructions and their visual referents. Language, cognition and neuroscience, 31(1), 129-144.

Josse, G., Joseph, S., Bertasi, E., & Giraud, A.-L. (2012). The Brain's Dorsal Route for Speech Represents Word Meaning: Evidence from Gesture. PLoS ONE, 7(9), e46108.

Kandylaki, K. D., Nagels, A., Tune, S., Kircher, T., Wiese, R., Schlesewsky, M., & Bornkessel-Schlesewsky, I. (2016). Predicting "when" in discourse engages the human dorsal auditory stream: An fMRI study using naturalistic stories. Journal of Neuroscience, 36(48), 12180-12191.

Kawawaki, D., Shibata, T., Goda, N., Doya, K., & Kawato, M. (2006). Anterior and superior lateral occipito-temporal cortex responsible for target motion prediction during overt and covert visual pursuit. Neuroscience research, 54(2), 112-123.

Keidel, J. L., Davis, P. M., Gonzalez-Diaz, V., Martin, C. D., & Thierry, G. (2013). How Shakespeare tempests the brain: neuroimaging insights. Cortex, 49(4), 913-919.

Kim, S. I., Hwang, S., & Lee, M. (2018). The benefits of negative yet informative feedback. PLoS One, 13(10), e0205183.

Klasen, M., Kenworthy, C. A., Mathiak, K. A., Kircher, T. T., & Mathiak, K. (2011). Supramodal representation of emotions. Journal of Neuroscience, 31(38), 13635-13643.

Kluger, D. S., & Schubotz, R. I. (2017). Strategic adaptation to non-reward prediction error qualities and irreducible uncertainty in fMRI. Cortex, 97, 32-48.

Koelsch, S., Fritz, T., Schulze, K., Alsop, D., & Schlaug, G. (2005). Adults and children processing music: an fMRI study. Neuroimage, 25(4), 1068-1076.

Koelsch, S., Gunter, T. C., Cramon, D. Y. V., Zysset, S., Lohmann, G., & Friederici, A. D. (2002). Bach speaks: a cortical "language-network" serves the processing of music. Neuroimage, 17(2), 956-966.

Koenen, L. R., Icenhour, A., Forkmann, K., Theysohn, N., Forsting, M., Bingel, U., & Elsenbruch, S. (2018). From anticipation to the experience of pain: the importance of visceral versus somatic pain modality in neural and behavioral responses to pain-predictive cues. Psychosomatic medicine, 80(9), 826-835.

Köhler, S., Bär, K. J., & Wagner, G. (2016). Differential involvement of brainstem noradrenergic and midbrain dopaminergic nuclei in cognitive control. Human brain mapping, 37(6), 2305-2318.

Kokonyei, G., Galambos, A., Edes, A. E., Kocsel, N., Szabo, E., Pap, D., ... & Juhasz, G. (2019). Anticipation and violated expectation of pain are influenced by trait rumination: An fMRI study. Cognitive, Affective, & Behavioral Neuroscience, 19, 56-72.

Krasovsky, A., Gilron, R., Yeshurun, Y., & Mukamel, R. (2014). Differentiating intended sensory outcome from underlying motor actions in the human brain. Journal of Neuroscience, 34(46), 15446-15454.

Krebs, R. M., Boehler, C. N., Egner, T., & Woldorff, M. G. (2011). The neural underpinnings of how reward associations can both guide and misguide attention. Journal of Neuroscience, 31(26), 9752-9759.

Krebs, R. M., Boehler, C. N., De Belder, M., & Egner, T. (2015). Neural conflict–control mechanisms improve memory for target stimuli. Cerebral Cortex, 25(3), 833-843.

Kristensen, L. B., Wang, L., Petersson, K. M., & Hagoort, P. (2013). The interface between language and attention: prosodic focus marking recruits a general attention network in spoken language comprehension. Cerebral Cortex, 23(8), 1836-1848.

Krug, M. K., & Carter, C. S. (2012). Proactive and reactive control during emotional interference and its relationship to trait anxiety. Brain research, 1481, 13-36.

Kudo, K., Miyazaki, M., Kimura, T., Yamanaka, K., Kadota, H., Hirashima, M., ... & Ohtsuki, T. (2004). Selective activation and deactivation of the human brain structures between speeded and precisely timed tapping responses to identical visual stimulus: an fMRI study. Neuroimage, 22(3), 1291-1301.

Langner, R., Kellermann, T., Boers, F., Sturm, W., Willmes, K., & Eickhoff, S. B. (2011). Modality-specific perceptual expectations selectively modulate baseline activity in auditory, somatosensory, and visual cortices. Cerebral cortex, 21(12), 2850-2862.

Laurienti, P. J., Wallace, M. T., Maldjian, J. A., Susi, C. M., Stein, B. E., & Burdette, J. H. (2003). Cross-modal sensory processing in the anterior cingulate and medial prefrontal cortices. Human brain mapping, 19(4), 213-223.

Leaver, A. M., Van Lare, J., Zielinski, B., Halpern, A. R., & Rauschecker, J. P. (2009). Brain activation during anticipation of sound sequences. Journal of Neuroscience, 29(8), 2477-2485.

Lee, D., Pruce, B., & Newman, S. D. (2014). The neural bases of argument structure processing revealed by primed lexical decision. Cortex, 57, 198-211.

Lee, H., & Noppeney, U. (2014). Temporal prediction errors in visual and auditory cortices. Current Biology, 24(8), R309-R310.

Leong, Y. C., Radulescu, A., Daniel, R., DeWoskin, V., & Niv, Y. (2017). Dynamic interaction between reinforcement learning and attention in multidimensional environments. Neuron, 93(2), 451-463.

Leube, D. T., Knoblich, G., Erb, M., & Kircher, T. T. (2003). Observing one's hand become anarchic: An fMRI study of action identification. Consciousness and cognition, 12(4), 597-608.

Li, S., Jiang, X., Yu, H., & Zhou, X. (2014). Cognitive empathy modulates the processing of pragmatic constraints during sentence comprehension. Social cognitive and affective neuroscience, 9(8), 1166-1174.

Lim, M., O'Grady, C., Cane, D., Goyal, A., Lynch, M., Beyea, S., & Hashmi, J. A. (2020). Threat prediction from schemas as a source of bias in pain perception. Journal of neuroscience, 40(7), 1538-1548.

Limanowski, J., & Blankenburg, F. (2015). Network activity underlying the illusory self-attribution of a dummy arm. Human Brain Mapping, 36(6), 2284-2304.

Limanowski, J., Kirilina, E., & Blankenburg, F. (2017). Neuronal correlates of continuous manual tracking under varying visual movement feedback in a virtual reality environment. NeuroImage, 146, 81-89.

Lüttke, C. S., Ekman, M., van Gerven, M. A., & de Lange, F. P. (2016). Preference for audiovisual speech congruency in superior temporal cortex. Journal of Cognitive Neuroscience, 28(1), 1-7.

Lyu, B., Ge, J., Niu, Z., Tan, L. H., & Gao, J. H. (2016). Predictive brain mechanisms in sound-to-meaning mapping during speech processing. Journal of Neuroscience, 36(42), 10813-10822.

Maffei, V., Giusti, M. A., Macaluso, E., Lacquaniti, F., & Viviani, P. (2015). Unfamiliar walking movements are detected early in the visual stream: an fMRI study. Cerebral Cortex, 25(8), 2022-2034.

Maffei, V., Indovina, I., Macaluso, E., Ivanenko, Y. P., Orban, G. A., & Lacquaniti, F. (2015). Visual gravity cues in the interpretation of biological movements: neural correlates in humans. Neuroimage, 104, 221-230.

Malekshahi, R., Seth, A., Papanikolaou, A., Mathews, Z., Birbaumer, N., Verschure, P. F., & Caria, A. (2016). Differential neural mechanisms for early and late prediction error detection. Scientific reports, 6(1), 24350.

Mancini, S., Quiñones, I., Molinaro, N., Hernandez-Cabrera, J. A., & Carreiras, M. (2017). Disentangling meaning in the brain: Left temporal involvement in agreement processing. Cortex, 86, 140-155.

Marini, F., Demeter, E., Roberts, K. C., Chelazzi, L., & Woldorff, M. G. (2016). Orchestrating proactive and reactive mechanisms for filtering distracting information: Brain-behavior relationships revealed by a mixed-design fMRI study. Journal of Neuroscience, 36(3), 988-1000.

Mastroberardino, S., Santangelo, V., & Macaluso, E. (2015). Crossmodal semantic congruence can affect visuo-spatial processing and activity of the fronto-parietal attention networks. Frontiers in integrative neuroscience, 9, 45.

Mayer, A. R., Franco, A. R., & Harrington, D. L. (2009). Neuronal modulation of auditory attention by informative and uninformative spatial cues. Human brain mapping, 30(5), 1652-1666.

Mayrhauser, L., Bergmann, J., Crone, J., & Kronbichler, M. (2014). Neural repetition suppression: evidence for perceptual expectation in object-selective regions. Frontiers in Human Neuroscience, 8, 225.

McAndrews, M. P., Girard, T. A., Wilkins, L. K., & McCormick, C. (2016). Semantic congruence affects hippocampal response to repetition of visual associations. Neuropsychologia, 90, 235-242.

Mead, L. A., Mayer, A. R., Bobholz, J. A., Woodley, S. J., Cunningham, J. M., Hammeke, T. A., & Rao, S. M. (2002). Neural basis of the Stroop interference task: response competition or selective attention?. Journal of the International Neuropsychological Society, 8(6), 735-742.

Mende-Siedlecki, P., & Todorov, A. (2016). Neural dissociations between meaningful and mere inconsistency in impression updating. Social Cognitive and Affective Neuroscience, 11(9), 1489-1500.

Mestres-Missé, A., Bazin, P. L., Trampel, R., Turner, R., & Kotz, S. A. (2014). Dorsomedial striatum involvement in regulating conflict between current and presumed outcomes. NeuroImage, 98, 159-167.

Mestres-Missé, A., Trampel, R., Turner, R., & Kotz, S. A. (2017). Uncertainty and expectancy deviations require cortico-subcortical cooperation. NeuroImage, 144, 23-34.

Michelon, P., Snyder, A. Z., Buckner, R. L., McAvoy, M., & Zacks, J. M. (2003). Neural correlates of incongruous visual information: an event-related fMRI study. Neuroimage, 19(4), 1612-1626.

Milham, M. P., Banich, M. T., & Barad, V. (2003). Competition for priority in processing increases prefrontal cortex's involvement in top-down control: an event-related fMRI study of the stroop task. Cognitive brain research, 17(2), 212-222.

Mill, R. D., Cavin, I., & O'Connor, A. R. (2015). Differentiating the functional contributions of resting connectivity networks to memory decision-making: fmri support for multistage control processes. Journal of cognitive neuroscience, 27(8), 1617-1632.

Moberget, T., Gullesen, E. H., Andersson, S., Ivry, R. B., & Endestad, T. (2014). Generalized role for the cerebellum in encoding internal models: evidence from semantic processing. Journal of Neuroscience, 34(8), 2871-2878.

Moris Fernandez, L., Macaluso, E., & Soto-Faraco, S. (2017). Audiovisual integration as conflict resolution: The conflict of the McGurk illusion. Human brain mapping, 38(11), 5691-5705.

Moris Fernández, L., Visser, M., Ventura-Campos, N., Ávila, C., & Soto-Faraco, S. (2015). Top-down attention regulates the neural expression of audiovisual integration. NeuroImage, 119, 272-285.

Nieuwland, M. S., Martin, A. E., & Carreiras, M. (2012). Brain regions that process case: evidence from Basque. Human brain mapping, 33(11), 2509-2520.

Noppeney, U., Josephs, O., Hocking, J., Price, C. J., & Friston, K. J. (2008). The effect of prior visual information on recognition of speech and sounds. Cerebral cortex, 18(3), 598-609.

Ochsner, K. N., Hughes, B., Robertson, E. R., Cooper, J. C., & Gabrieli, J. D. (2009). Neural systems supporting the control of affective and cognitive conflicts. Journal of cognitive neuroscience, 21(9), 1841-1854.

Ogawa, K., Inui, T., & Sugio, T. (2007). Neural correlates of state estimation in visually guided movements: an event-related fMRI study. Cortex, 43(3), 289-300.

Okada, K., Matchin, W., & Hickok, G. (2018). Neural evidence for predictive coding in auditory cortex during speech production. Psychonomic bulletin & review, 25, 423-430.

Ondobaka, S., de Lange, F. P., Wittmann, M., Frith, C. D., & Bekkering, H. (2015). Interplay between conceptual expectations and movement predictions underlies action understanding. Cerebral cortex, 25(9), 2566-2573.

O'Reilly, J. X., Schüffelgen, U., Cuell, S. F., Behrens, T. E., Mars, R. B., & Rushworth, M. F. (2013). Dissociable effects of surprise and model update in parietal and anterior cingulate cortex. Proceedings of the National Academy of Sciences, 110(38), E3660-E3669.

Osnes, B., Hugdahl, K., Hjelmervik, H., & Specht, K. (2012). Stimulus expectancy modulates inferior frontal gyrus and premotor cortex activity in auditory perception. Brain and language, 121(1), 65-69.

Park, H. R., Kostandyan, M., Boehler, C. N., & Krebs, R. M. (2019). Winning smiles: Signalling reward by overlapping and non-overlapping emotional valence differentially affects performance and neural activity. Neuropsychologia, 122, 28-37.

Pazen, M., Uhlmann, L., van Kemenade, B. M., Steinsträter, O., Straube, B., & Kircher, T. (2020). Predictive perception of self-generated movements: commonalities and differences in the neural processing of tool and hand actions. NeuroImage, 206, 116309.

Peeters, D., Snijders, T. M., Hagoort, P., & Özyürek, A. (2017). Linking language to the visual world: Neural correlates of comprehending verbal reference to objects through pointing and visual cues. Neuropsychologia, 95, 21-29.

Pichon, S., Guex, R., & Vuilleumier, P. (2016). Influence of temporal expectations on response priming by subliminal faces. PLoS One, 11(10), e0164613.

Potgieser, A. R., & de Jong, B. M. (2016). Visuomotor dissociation in cerebral scaling of size. PloS one, 11(3), e0151484.

Quiñones, I., Molinaro, N., Mancini, S., Hernández-Cabrera, J. A., & Carreiras, M. (2014). Where agreement merges with disagreement: fMRI evidence of subject–verb integration. NeuroImage, 88, 188-201.

Raettig, T., Frisch, S., Friederici, A. D., & Kotz, S. A. (2010). Neural correlates of morphosyntactic and verb-argument structure processing: An EfMRI study. Cortex, 46(5), 613-620.

Rahnev, D., Lau, H., & De Lange, F. P. (2011). Prior expectation modulates the interaction between sensory and prefrontal regions in the human brain. Journal of Neuroscience, 31(29), 10741-10748.

Ramnani, N., & Miall, R. C. (2003). Instructed delay activity in the human prefrontal cortex is modulated by monetary reward expectation. Cerebral Cortex, 13(3), 318-327.

Ran, G., Chen, X., Cao, X., & Zhang, Q. (2016). Prediction and unconscious attention operate synergistically to facilitate stimulus processing: An fMRI study. Consciousness and cognition, 44, 41-50.

Reichert, J. L., Ninaus, M., Schuehly, W., Hirschmann, C., Bagga, D., & Schöpf, V. (2017). Functional brain networks during picture encoding and recognition in different odor contexts. Behavioural Brain Research, 333, 98-108.

Reicherts, P., Wiemer, J., Gerdes, A. B., Schulz, S. M., Pauli, P., & Wieser, M. J. (2017). Anxious anticipation and pain: the influence of instructed vs conditioned threat on pain. Social cognitive and affective neuroscience, 12(4), 544-554.

Richter, D., & de Lange, F. P. (2019). Statistical learning attenuates visual activity only for attended stimuli. elife, 8, e47869.

Rilling, J. K., Sanfey, A. G., Aronson, J. A., Nystrom, L. E., & Cohen, J. D. (2004). Opposing BOLD responses to reciprocated and unreciprocated altruism in putative reward pathways. Neuroreport, 15(16), 2539-2243.

Roberts, K. L., & Hall, D. A. (2008). Examining a supramodal network for conflict processing: a systematic review and novel functional magnetic resonance imaging data for related visual and auditory stroop tasks. Journal of cognitive neuroscience, 20(6), 1063-1078.

Robertson, B. D., Hiebert, N. M., Seergobin, K. N., Owen, A. M., & MacDonald, P. A. (2015). Dorsal striatum mediates cognitive control, not cognitive effort per se, in decision-making: An event-related fMRI study. NeuroImage, 114, 170-184.

Roelofs, A., Van Turennout, M., & Coles, M. G. (2006). Anterior cingulate cortex activity can be independent of response conflict in Stroop-like tasks. Proceedings of the National Academy of Sciences, 103(37), 13884-13889.

Rothermich, K., & Kotz, S. A. (2013). Predictions in speech comprehension: fMRI evidence on the meter–semantic interface. NeuroImage, 70, 89-100.

Russo, A. G., De Martino, M., Mancuso, A., Iaconetta, G., Manara, R., Elia, A., ... & Esposito, F. (2020). Semantics-weighted lexical surprisal modeling of naturalistic functional MRI time-series during spoken narrative listening. Neuroimage, 222, 117281.

Sacheli, L. M., Verga, C., Arcangeli, E., Banfi, G., Tettamanti, M., & Paulesu, E. (2019). How task interactivity shapes action observation. Cerebral Cortex, 29(12), 5302-5314.

Säfström, D., & Domellöf, E. (2018). Brain activations supporting linking of action phases in a sequential manual task. Neuroimage, 172, 608-619.

Sahyoun, C., Floyer-Lea, A., Johansen-Berg, H., & Matthews, P. M. (2004). Towards an understanding of gait control: brain activation during the anticipation, preparation and execution of foot movements. Neuroimage, 21(2), 568-575.

Sali, A. W., Jiang, J., & Egner, T. (2020). Neural mechanisms of strategic adaptation in attentional flexibility. Journal of cognitive neuroscience, 32(5), 989-1008.

Sayalı, C., & Badre, D. (2021). Neural systems underlying the learning of cognitive effort costs. Cognitive, Affective, & Behavioral Neuroscience, 21, 698-716.

Saygin, A. P., Chaminade, T., Ishiguro, H., Driver, J., & Frith, C. (2012). The thing that should not be: predictive coding and the uncanny valley in perceiving human and humanoid robot actions. Social cognitive and affective neuroscience, 7(4), 413-422.

Schiffer, A. M., & Schubotz, R. I. (2011). Caudate nucleus signals for breaches of expectation in a movement observation paradigm. Frontiers in Human Neuroscience, 5, 38.

Schiffer, A. M., Ahlheim, C., Ulrichs, K., & Schubotz, R. I. (2013). Neural changes when actions change: adaptation of strong and weak expectations. Human brain mapping, 34(7), 1713-1727.

Schiffler, B. C., Almeida, R., Granqvist, M., & Bengtsson, S. L. (2016). Memory-reliant post-error slowing is associated with successful learning and fronto-occipital activity. Journal of Cognitive Neuroscience, 28(10), 1539-1552.

Schneider, M., Leuchs, L., Czisch, M., Sämann, P. G., & Spoormaker, V. I. (2018). Disentangling reward anticipation with simultaneous pupillometry/fMRI. NeuroImage, 178, 11-22.

Schuster, S., Himmelstoss, N. A., Hutzler, F., Richlan, F., Kronbichler, M., & Hawelka, S. (2021). Cloze enough? Hemodynamic effects of predictive processing during natural reading. NeuroImage, 228, 117687.

Schuwerk, T., Döhnel, K., Sodian, B., Keck, I. R., Rupprecht, R., & Sommer, M. (2014). Functional activity and effective connectivity of the posterior medial prefrontal cortex during processing of incongruent mental states. Human brain mapping, 35(7), 2950-2965.

Seger, C. A., Spiering, B. J., Sares, A. G., Quraini, S. I., Alpeter, C., David, J., & Thaut, M. H. (2013). Corticostriatal contributions to musical expectancy perception. Journal of cognitive neuroscience, 25(7), 1062-1077.

Seid-Fatemi, A., & Tobler, P. N. (2015). Efficient learning mechanisms hold in the social domain and are implemented in the medial prefrontal cortex. Social Cognitive and Affective Neuroscience, 10(5), 735-743.

Seidel, E. M., Pfabigan, D. M., Hahn, A., Sladky, R., Grahl, A., Paul, K., ... & Lamm, C. (2015). Uncertainty during pain anticipation: the adaptive value of preparatory processes. Human Brain Mapping, 36(2), 744-755.

Seymour, B., O'doherty, J. P., Koltzenburg, M., Wiech, K., Frackowiak, R., Friston, K., & Dolan, R. (2005). Opponent appetitive-aversive neural processes underlie predictive learning of pain relief. Nature neuroscience, 8(9), 1234-1240.

Sharvit, G., Corradi-Dell'Acqua, C., & Vuilleumier, P. (2018). Modality-specific effects of aversive expectancy in the anterior insula and medial prefrontal cortex. Pain, 159(8), 1529-1542.

Sherman, M. T., Seth, A. K., & Kanai, R. (2016). Predictions shape confidence in right inferior frontal gyrus. Journal of Neuroscience, 36(40), 10323-10336.

Sheu, Y. S., & Desmond, J. E. (2021). Cerebro-cerebellar response to sequence violation in a cognitive task: an fMRI study. The Cerebellum, 1-13.

Shin, Y. S., Kim, H. Y., & Han, S. (2014). Neural correlates of social perception on response bias. Brain and cognition, 88, 55-64.

Singh, L., Schüpbach, L., Moser, D. A., Wiest, R., Hermans, E. J., & Aue, T. (2020). The effect of optimistic expectancies on attention bias: Neural and behavioral correlates. Scientific reports, 10(1), 6495.

Sitnikova, T., Rosen, B. R., Lord, L. D., & West, W. C. (2014). Understanding human original actions directed at real-world goals: The role of the lateral prefrontal cortex. NeuroImage, 103, 91-105.

Smith, D. V., Davis, B., Niu, K., Healy, E. W., Bonilha, L., Fridriksson, J., ... & Rorden, C. (2010). Spatial attention evokes similar activation patterns for visual and auditory stimuli. Journal of cognitive neuroscience, 22(2), 347-361.

Söderström, P., Horne, M., Mannfolk, P., van Westen, D., & Roll, M. (2018). Rapid syntactic pre-activation in Broca's area: Concurrent electrophysiological and haemodynamic recordings. Brain Research, 1697, 76-82.

Sosic-Vasic, Z., Ulrich, M., Ruchsow, M., Vasic, N., & Grön, G. (2012). The modulating effect of personality traits on neural error monitoring: evidence from event-related FMRI.

Stanley, J., & Miall, R. C. (2007). Functional activation in parieto-premotor and visual areas dependent on congruency between hand movement and visual stimuli during motor-visual priming. Neuroimage, 34(1), 290-299.

Stanley, D. A. (2016). Getting to know you: general and specific neural computations for learning about people. Social Cognitive and Affective Neuroscience, 11(4), 525-536.

Stefanics, G., Stephan, K. E., & Heinzle, J. (2019). Feature-specific prediction errors for visual mismatch. Neuroimage, 196, 142-151.

Stevens, M. C., Calhoun, V. D., & Kiehl, K. A. (2005). Hemispheric differences in hemodynamics elicited by auditory oddball stimuli. Neuroimage, 26(3), 782-792.

Stewart, H. J., Shen, D., Sham, N., & Alain, C. (2020). Involuntary orienting and conflict resolution during auditory attention: The role of ventral and dorsal streams. Journal of Cognitive Neuroscience, 32(10), 1851-1863.

Suzuki, S., Harasawa, N., Ueno, K., Gardner, J. L., Ichinohe, N., Haruno, M., ... & Nakahara, H. (2012). Learning to simulate others' decisions. Neuron, 74(6), 1125-1137.

Taylor, S. F., Kornblum, S., Lauber, E. J., Minoshima, S., & Koeppe, R. A. (1997). Isolation of specific interference processing in the Stroop task: PET activation studies. Neuroimage, 6(2), 81-92.

Tesink, C. M., Petersson, K. M., Van Berkum, J. J., Van den Brink, D., Buitelaar, J. K., & Hagoort, P. (2009). Unification of speaker and meaning in language comprehension: An fMRI study. Journal of Cognitive Neuroscience, 21(11), 2085-2099.

Thioux, M., & Keysers, C. (2015). Object visibility alters the relative contribution of ventral visual stream and mirror neuron system to goal anticipation during action observation. NeuroImage, 105, 380-394.

Thomas, R. M., De Sanctis, T., Gazzola, V., & Keysers, C. (2018). Where and how our brain represents the temporal structure of observed action. NeuroImage, 183, 677-697.

Thompson, R., & Duncan, J. (2009). Attentional modulation of stimulus representation in human fronto-parietal cortex. Neuroimage, 48(2), 436-448.

Thornton, M. A., Weaverdyck, M. E., & Tamir, D. I. (2019). The social brain automatically predicts others' future mental states. Journal of Neuroscience, 39(1), 140-148.

Tillmann, B., Janata, P., & Bharucha, J. J. (2003). Activation of the inferior frontal cortex in musical priming. Cognitive Brain Research, 16(2), 145-161.

Tillmann, B., Koelsch, S., Escoffier, N., Bigand, E., Lalitte, P., Friederici, A. D., & von Cramon, D. Y. (2006). Cognitive priming in sung and instrumental music: activation of inferior frontal cortex. Neuroimage, 31(4), 1771-1782.

Tipper, C. M., Signorini, G., & Grafton, S. T. (2015). Body language in the brain: constructing meaning from expressive movement. Frontiers in human neuroscience, 9, 450.

Trempler, I., Schiffer, A. M., El-Sourani, N., Ahlheim, C., Fink, G. R., & Schubotz, R. I. (2017). Frontostriatal contribution to the interplay of flexibility and stability in serial prediction. Journal of cognitive neuroscience, 29(2), 298-309.

Tune, S., Schlesewsky, M., Nagels, A., Small, S. L., & Bornkessel-Schlesewsky, I. (2016). Sentence understanding depends on contextual use of semantic and real world knowledge. NeuroImage, 136, 10-25.

Uhlmann, L., Pazen, M., van Kemenade, B. M., Steinsträter, O., Harris, L. R., Kircher, T., & Straube, B. (2020). Seeing your own or someone else's hand moving in accordance with your action: The neural interaction of agency and hand identity. Human brain mapping, 41(9), 2474-2489.

van Assche, M., Kebets, V., Vuilleumier, P., & Assal, F. (2016). Functional dissociations within posterior parietal cortex during scene integration and viewpoint changes. Cerebral Cortex, 26(2), 586-598.

van Atteveldt, N. M., Formisano, E., Goebel, R., & Blomert, L. (2007). Top–down task effects overrule automatic multisensory responses to letter–sound pairs in auditory association cortex. Neuroimage, 36(4), 1345-1360.

van de Meerendonk, N., Rueschemeyer, S. A., & Kolk, H. H. (2013). Language comprehension interrupted: Both language errors and word degradation activate Broca's area. Brain and Language, 126(3), 291-301.

Vanyukov, P. M., Hallquist, M. N., Delgado, M., Szanto, K., & Dombrovski, A. Y. (2019). Neurocomputational mechanisms of adaptive learning in social exchanges. Cognitive, Affective, & Behavioral Neuroscience, 19, 985-997.

Visalli, A., Capizzi, M., Ambrosini, E., Mazzonetto, I., & Vallesi, A. (2019). Bayesian modeling of temporal expectations in the human brain. Neuroimage, 202, 116097.

Volz, K. G., Schubotz, R. I., & von Cramon, D. Y. (2003). Predicting events of varying probability: uncertainty investigated by fMRI. Neuroimage, 19(2), 271-280.

Vossel, S., Mathys, C., Stephan, K. E., & Friston, K. J. (2015). Cortical coupling reflects Bayesian belief updating in the deployment of spatial attention. Journal of Neuroscience, 35(33), 11532-11542.

Walter, H., Kausch, A., Dorfschmidt, L., Waller, L., Chinichian, N., Veer, I., ... & Kruschwitz, J. D. (2020). Self-control and interoception: Linking the neural substrates of craving regulation and the prediction of aversive interoceptive states induced by inspiratory breathing restriction. Neuroimage, 215, 116841.

Weber, K., Micheli, C., Ruigendijk, E., & Rieger, J. W. (2019). Sentence processing is modulated by the current linguistic environment and a priori information: An fMRI study. Brain and Behavior, 9(7), e01308.

Wessel, J. R., Danielmeier, C., Morton, J. B., & Ullsperger, M. (2012). Surprise and error: common neuronal architecture for the processing of errors and novelty. Journal of Neuroscience, 32(22), 7528-7537.

Willems, R. M., Frank, S. L., Nijhof, A. D., Hagoort, P., & Van den Bosch, A. (2016). Prediction during natural language comprehension. Cerebral Cortex, 26(6), 2506-2516.

Wittmann, B. C., Schott, B. H., Guderian, S., Frey, J. U., Heinze, H. J., & Düzel, E. (2005). Reward-related FMRI activation of dopaminergic midbrain is associated with enhanced hippocampus-dependent long-term memory formation. Neuron, 45(3), 459-467.

Wolf, D., Schock, L., Bhavsar, S., Demenescu, L. R., Sturm, W., & Mathiak, K. (2014). Emotional valence and spatial congruency differentially modulate crossmodal processing: an fMRI study. Frontiers in Human Neuroscience, 8, 659.

Wright, M., Bishop, D. T., Jackson, R., & Abernethy, B. (2013). Brain regions concerned with the identification of deceptive soccer moves by higher-skilled and lower-skilled players. Frontiers in human neuroscience, 7, 851.

Yomogida, Y., Sugiura, M., Sassa, Y., Wakusawa, K., Sekiguchi, A., Fukushima, A., ... & Kawashima, R. (2010). The neural basis of agency: an fMRI study. Neuroimage, 50(1), 198-207.

Zhao, S., Li, C., Uono, S., Yoshimura, S., & Toichi, M. (2017). Human cortical activity evoked by contextual processing in attentional orienting. Scientific reports, 7(1), 2962.

Zuanazzi, A., & Noppeney, U. (2019). Distinct neural mechanisms of spatial attention and expectation guide perceptual inference in a multisensory world. Journal of Neuroscience, 39(12), 2301-2312.
