# Supplementary material for: Comprehensive investigation of predictive processing: A cross‐ and within‐cognitive domains fMRI meta‐analytic approach
Source: Hum Brain Mapp. 2024 Aug 21;45(12):e26817. doi: 10.1002/hbm.26817 (PMC11339134; doi:10.1002/hbm.26817)
Supplement: Supplementary file 4 — Data S4. [file HBM-45-e26817-s001.docx]

**Supplementary Information D**

**Checklist for neuroimaging meta-analysis (Muller et al. 2018)**

| The research question is specifically defined | **YES**:  Aim 1: replicate Siman-Tov (2019) meta-analysis using Ginger ALE and SDM softwares;  Aim 2: perform separate meta-analyses on several cognitive domains which used tasks aimed at investigating predictive processes. For each cognitive domain, the contrast selected will be the following one:   - cognitive function involving prediction > control conditions devoid of prediction   Aim 3: explore the presence of overlap between the networks emerged from the meta-analyses, using the following approach:   - cognitive domain 1 (prediction vs no prediction) ∩ cognitive domain 2 (prediction vs no prediction) ∩ cognitive domain 3 (prediction vs no prediction) ∩ etc.   Aim 4: compare visually the network emerged from Siman-Tov replication and the one that will emerge from this study by means of a simple overlap of ALE and SDM maps;  Aim 5: adopt the functional characterization approach to identify which psychological/cognitive processes are correlated with the regions identified by the meta-analyses;  AIm 6: search for a functional gradient organization. |
| --- | --- |
| The literature search was systematic | **YES (PRISMA guidelines used)**, it includes the following keywords in the following databases:  **Keywords**:  The search algorithm has the following structure:  (neuroimaging AND prediction AND cognitive domain NOT exclusion criteria).  Note that the keywords related to neuroimaging, prediction and exclusion criteria are the same for each cognitive domain, for this reason, they will be presented under the following “general keywords” section.  General Keywords:  **NeuroImaging:** (((neuroimaging [Title/Abstract] OR fMRI[Title/Abstract] OR functional MRI[Title/Abstract] OR magnetic resonance imaging[Title/Abstract] OR PET[Title/Abstract]) NOT (DTI[Title/Abstract] OR Diffusion tensor Imaging[Title/Abstract] OR machine learning[Title/Abstract] OR structural[Title/Abstract] OR tDCS[Title/Abstract] OR TMS[Title/Abstract] OR EEG[Title/Abstract] OR intracranial[Title/Abstract]))  **Prediction:** (prediction[Title/Abstract] OR expectancy[Title/Abstract] OR expectation[Title/Abstract] OR anticipation[Title/Abstract] OR unexpected[Title/Abstract] OR surpris*[Title/Abstract] OR prediction error[Title/Abstract] OR incongruent[Title/Abstract] OR irregular[Title/Abstract] OR violat*[Title/Abstract] OR mismatch[Title/Abstract] OR anticipat*[Title/Abstract])  **Exclusion criteria:** (disease[Title/Abstract] OR disorder[Title/Abstract] OR pathology[Title/Abstract] OR psychiat*[Title/Abstract] OR stroke[Title/Abstract] OR neurologic*[Title/Abstract] OR Alzheimer[Title/Abstract] OR Parkinson[Title/Abstract] OR depression[Title/Abstract] OR schizophrenia[Title/Abstract] OR dementia[Title/Abstract] OR neglect[Title/Abstract] OR drug*[Title/Abstract] OR brain injury[Title/Abstract] OR surgery[Title/Abstract] OR surgical[Title/Abstract] OR damag*[Title/Abstract] OR animal*[Title/Abstract] OR infants[Title/Abstract] OR adolescents[Title/Abstract] OR older[Title/Abstract] OR elderly[Title/Abstract] OR child*[Title/Abstract] OR phantom[Title/Abstract] OR rats[Title/Abstract] OR aphasi*[Title/Abstract] OR sign[Title/Abstract] OR adhd[Title/Abstract] OR deficit*[Title/Abstract] OR patient*[Title/Abstract] OR single-case[Title/Abstract] OR rodent*[Title/Abstract] OR review[Publication Type] OR meta-analysis[Publication Type] OR developmental[Title/Abstract] OR infants[Title/Abstract] OR surprisingly[Title/Abstract] OR unexpected results[Title/Abstract] OR unexpectedly[Title/Abstract] OR treatment[Title/Abstract] OR training[Title/Abstract] OR longitudinal[Title/Abstract])  Keywords specific for each cognitive function:  SOCIAL COGNITION: (social cognition[Title/Abstract] OR theory of mind[Title/Abstract] OR mentalizing[Title/Abstract] OR mindreading[Title/Abstract] OR mind-reading[Title/Abstract] OR mind reading[Title/Abstract] OR ToM[Title/Abstract] OR social learning[Title/Abstract])  LANGUAGE: (language[Title/Abstract] OR semantic*[Title/Abstract] OR syntactic[Title/Abstract] OR pragmatic*[Title/Abstract])  MUSIC: (music*[Title/Abstract] OR harmonic[Title/Abstract] OR melodic[Title/Abstract] OR rhythmic[Title/Abstract])  ATTENTION: (attention[Title/Abstract])  MOTOR: (motor planning[Title/Abstract] OR movement[Title/Abstract] OR kinematic*[Title/Abstract])  MEMORY: (prospective memory[Title/Abstract] OR episodic memory[Title/Abstract] OR semantic memory[Title/Abstract] OR procedural memory[Title/Abstract] OR long-term memory[Title/Abstract] OR working memory[Title/Abstract] OR short-term memory[Title/Abstract])  INTEROCEPTION: (interocept*[Title/Abstract] OR internal attention[Title/Abstract] OR heart beat[Title/Abstract] OR heart-beat[Title/Abstract] OR internal states[Title/Abstract] OR visceral states[Title/Abstract] OR pain[Title/Abstract] OR bodily changes[Title/Abstract])  EXECUTIVE FUNCTIONS: (executive function*[Title/Abstract] OR cognitive flexibility[Title/Abstract] OR perseverat*[Title/Abstract] OR cognitive control[Title/Abstract] OR error-monitoring[Title/Abstract] OR error monitoring[Title/Abstract] OR error detection[Title/Abstract] OR cognitive conflict[Title/Abstract])  PAIN: pain[Title/Abstract]  **Databases**: PubMed, embase, psychinfo, “related article” function in pubmed, previous meta-analyses, reference within the selected literature. Only articles with human participants, with English as language of reference and that have been peer-reviewed have been included. |
| Detailed inclusion and exclusion criteria are included | **YES**, and reason for non standard criterion were:  Standard criteria applied: only whole brain experiment included; only studies reporting results in a standardized coordinate space were included.  Non standard criteria applied:   - studies that used fMRI or PET 🡪 criterion decided in order to maximize the power of the meta-analysis and in order to exclude studies with structural MRI; - studies analyzing the data using univariate approach that revealed localized increased activation were included 🡪 criterion decided to exclude papers that analyzed data using machine learning, whose results have a slightly different meaning; and to exclude papers using functional connectivity techniques, as we are not interested in connectivity; - studies with sample size of at least 5 participants (per group) were included 🡪 criterion decided according with previous coordinate based meta-analysis in order to reduce the likelihood to include studies presenting false positives; - studies were included only if they are performed on healthy individuals 🡪 criterion decided in accordance with the research question (to investigate the neural basis of normal spatial or temporal processing); - only studies reporting the contrast “specific cognitive domain” (which includes prediction) > control condition devoid of the prediction component were included 🡪 criterion decided to increase the specificity of the meta-analysis. |
| Sample overlap was taken into account | **YES**, using the following method:  For each paper, only the contrast that most strongly reflects the process that the meta-analysis aims to investigate has been selected. Two contrasts for a single paper will be selected only if authors run the analysis on two independent samples, and this is clearly stated in the original paper. |
| All experiments use the same search coverage (state how brain coverage is assessed and how small volume corrections and conjunctions are taken into account) | **YES**, the search coverage is the following:  Whole brain.  If a study reported whole brain + ROI analysis, the whole brain analysis only has been included in the meta-analysis; if a study reported the ROI analysis only, the study was excluded from the meta-analysis in accordance with the inclusion/exclusion criteria.  Paper applying small volume correction or hidden ROI (i.e. where fMRI data were not acquired on the whole brain) will be excluded. |
| Studies are converted to a common reference space | **YES**, using the following conversion:  Tailarach coordinates were reported into MNI space using a linear transformation as implemented in the most updated Ginger ALE version at the time of the analysis. |
| Data extraction have been conducted by two investigators (ideal case) or double checked by the same investigator (state how double checking was performed) | **YES**, the following authors:  CC, FM, RP, SL will check inclusion criteria;  CC, FM, RP, SL will extract coordinates;  CC, FM, RP, SL will extract other info: Number of subjects included, Type of task (cognitive function involved); task (specific task); contrast performed; coordinate system; associated statistic (t value, z score); p value criteria (corrected, uncorrected), etc.;  CS will randomly double-check the following data: extracted coordinates, inclusion criteria, additional info.  In case of conflictual opinion on data extraction between the initial rater and CS, and additional author (GA) will solve the conflict. |
| The paper includes a table with at least the references, basic study description (e.g. for fMRI task: stimuli), contrasts and basic sample descriptions (e.g. size, mean age and gender distribution, etc) of the included studies, source of information, reference space | **YES** and the paper will also include the following data:  Study reference; Number of subjects included; task (specific task); task modality; type of stimuli; specific cognitive domain; predictive phenomena; contrast performed; coordinate system; associated statistic (t value, z score); p value criteria (corrected, uncorrected);  Please note that this table could also not be enclosed within the paper. In this case it will be available within the supplementary information as an excel database. |
| The study protocol was previously registered and all analyses planned beforehand, including the methods and parameters used for inference, correction for multiple testing, etc. | **YES**:   1. prior to star data extraction, the study will be pre-registered in OSF; 2. the analyses have been planned before starting the literature search; 3. if we will run any non-planned or non-prespecified analysis, this information will be clearly present in the paper as a deviation from pre-registration; 4. The meta-analysis will use the default methods and parameters of the software with no exceptions. |
| The meta-analysis includes diagnostics | Effect size, heterogeneity of data and publication bias will be calculated by means of SDM software.  Depending on the final number of included studies, meta-analyses could be repeated, removing some studies to harmonize the number of studies on each cognitive domain.  In case many studies for a specific cognitive domain come from the same laboratory, analyses will be repeated keeping only one study for each laboratory, as in previous meta-analyses. |
